# Gene Expression in Embryos From Norwegian Red Bulls With High or Low Non Return Rate: An RNA-Seq Study of in vivo-Produced Single Embryos

**DOI:** 10.3389/fgene.2021.780113

**Published:** 2022-01-14

**Authors:** Sofia Diaz-Lundahl, Arvind Y.M. Sundaram, Per Gillund, Gregor Duncan Gilfillan, Ingrid Olsaker, Anette Krogenæs

**Affiliations:** ^1^ Department of Production Animal Clinical Sciences, Faculty of Veterinary Medicine, Norwegian University of Life Sciences, Ås, Norway; ^2^ Department of Medical Genetics, Oslo University Hospital and University of Oslo, Oslo, Norway; ^3^ Geno Breeding and AI Association, Hamar, Norway; ^4^ Department of Preclinical Sciences and Pathology, Faculty of Veterinary Medicine, Norwegian University of Life Sciences, Ås, Norway

**Keywords:** bull fertility, paternal influence, Norwegian Red bulls, RNA-seq, gene expression, bovine preimplantation embryos, embryo mortality, *in vivo* produced embryos

## Abstract

During the last decade, paternal effects on embryo development have been found to have greater importance than previously believed. In domestic cattle, embryo mortality is an issue of concern, causing huge economical losses for the dairy cattle industry. In attempts to reveal the paternal influence on embryo death, recent approaches have used transcriptome profiling of the embryo to find genes and pathways affected by different phenotypes in the bull. For practical and economic reasons, most such studies have used *in vitro* produced embryos. The aim of the present study was to investigate the differences in the global transcriptome of *in vivo* produced embryos, derived from sires with either high or low field fertility measured as the non-return rate (NRR) on day 56 after first AI of the inseminated cows. Superovulated heifers (*n* = 14) in the age span of 12–15 months were artificially inseminated with semen from either high fertility (*n* = 6) or low fertility (*n* = 6) bulls. On day seven after insemination, embryos were retrieved through uterine flushing. Embryos with first grade quality and IETS stage 5 (early blastocyst), 6 (blastocyst) or 7 (expanded blastocyst) were selected for further processing. In total, RNA extracted from 24 embryos was sequenced using Illumina sequencing, followed by differential expression analysis and gene set enrichment analysis. We found 62 genes differentially expressed between the two groups (adj.*p*-value<0.05), of which several genes and their linked pathways could explain the different developmental capacity. Transcripts highly expressed in the embryos from low fertility bulls were related to sterol metabolism and terpenoid backbone synthesis, while transcripts highly expressed in the high fertility embryos were linked to anti-apoptosis and the regulation of cytokine signaling. The leukocyte transendothelial migration and insulin signaling pathways were associated with enrichments in both groups. We also found some highly expressed transcripts in both groups which can be considered as new candidates in the regulation of embryo development. The present study is an important step in defining the paternal influence in embryonic development. Our results suggest that the sire’s genetic contribution affects several important processes linked to pre-and peri implantation regulation in the developing embryo.

## Introduction

Embryo mortality is an issue of concern in dairy cattle breeding, being the most common cause for failed pregnancy (Diskin et al., 2006), with negative consequences for milk and food production and corresponding economic impact. The majority of embryo mortality occurs within 16 days from breeding, and probably within the first 8 days for cows with a high milk yield (Diskin et al., 2016). A former commonly accepted theory stated that early embryo development was exclusively regulated by the mother, based on the fact that the female gamete is much larger than the male gamete and consequently had the capacity to house the necessary regulating factors such as transcripts and proteins (Immler, 2018). As it was revealed that breeding for high milk yield could have an inverse effect on reproductive outcome, complying with the earlier decline in fertility observed worldwide, the cow became the main target for studies related to embryo mortality (Kropp et al., 2014). In contrast, previous investigations on the father’s contribution to fertility mainly focused on morphological assessments of the spermatozoa’s ability to reach and fertilize the oocyte (Moldenhauer et al., 2003; Daigneault, 2020). More recent evidence demonstrates that both parents contribute to embryo programming, through genetic and epigenetic components, and *via* RNAs and proteins directly deposited within the zygote (Gross et al., 2019; Daigneault, 2020; Wu and Sirard, 2020). Thus, both male and female fertility can be defined as the capacity of fertilization and continued embryo and fetal development until birth. Consequently, the contribution from both parents can be responsible for embryo death. Separate investigation of male fertility is crucial, as the correlation of genetic progress in the fertility of the bull and the cow is low (Taylor et al., 2018).

Recent advances in biotechnology have initiated an understanding of genetic control of the embryo, investigating different levels of genomics and epigenomics through single-embryo analysis at different developmental stages and qualities (Huang et al., 2010; Graf et al., 2014; Kropp et al., 2017). Jiang et al. (2014) investigated *in vivo* produced embryos from three different species and found that the bovine embryo is a better model for human embryonic development than the mouse embryo, implying that studies on the bovine embryo are highly relevant beyond the field of veterinary science. In cattle, the major embryonic gene activation (EGA) occurs at the 8-cell stage (Graf et al., 2014). Studies have revealed that the 2-4 cell bovine embryo consists of both maternal-specific and paternal-specific transcripts (Gross et al., 2019). These transcripts have the potential to affect embryonic development, both at that specific stage and in later developmental stages. It has also been demonstrated that the father contributes on the epigenomic level, with mechanisms such as chromatin structure alterations and DNA-methylation differing between high and low fertility bulls (Kropp et al., 2017), affecting the fate of gene transcription in the embryo. The exact function of paternally delivered transcripts or their regulation of genes that control embryo development remains largely unclear.

Norwegian Red (NR) is the main dairy breed in Norway. The breeding program has had a strong emphasis on fertility and health since the 1970s. The Norwegian Dairy Herd Recording System is well-established and includes information on fertility outcome that can be used for investigations related to bull fertility. The breeding strategy for NR was recently changed from progeny testing to genomic selection, which results in a faster breeding progress. The bulls are now in semen production at an earlier age, and identification of reliable markers for the bull fertility is of increased interest. By comparing different phenotypes in the father with the outcome in the embryo, one can reveal genes and pathways that are affected by the bull’s contribution, which in a longer perspective could support the prediction of bull fertility. At the blastocyst stage, Kropp and colleagues demonstrated that *in vitro* produced embryos from bulls of different fertility had different gene expression (Kropp et al., 2017). Another study investigated the transcriptome of IVF blastocysts derived from the same father animal at either 10, 12 or 16 months of age. Using microarray data, they found several genes to be differentially expressed depending on the age of the father (Wu et al., 2020). *In vitro* production of embryos offers a valuable research tool with a high level of feasibility and accuracy. However, even under detailed control, an *in vitro* system may affect or alter the gene expression through stressors that are unnatural for an embryo. Hence, theories established by *in vitro* studies need to be considered in an *in vivo* approach. The current study aimed to explore the differences in gene expression, on a whole transcriptomic scale, of *in vivo* produced single blastocyst embryos derived from two groups of Norwegian red bulls with high or low non-return rate.

## Material and Methods

### Animals

The present study used frozen semen from 12 NR bulls, divided into two groups based on fertility. The bulls were selected from a database of 470 NR bulls, born between 2010 and 2014, all with at least 500 registered first inseminations (AI). Fertility was recorded as the non-return rate (NRR) at 56 days after first AI of the inseminated cows, and varied from 49.3 to 80.5 with an average of 72.5 (s.e. = 3.5). The 12 selected bulls had a record of 661–901 first artificial inseminations and represented the highest and lowest fertility among all registrations. Bulls in the high fertility (HF) group had a NRR of 78.7–80.5 (*n* = 6), while the low fertility group (LF) had an NRR of 49.3–62.1 (*n* = 6). The reason for the difference in fertility was not known, and the semen was no longer in commercial use. The semen had passed standard testing requirements performed by Geno SA^1^, the breeding organization for NR cattle, before commercial use; Macroscopic evaluation, a concentration threshold of 390 million cells per ml, at least 70 and 50% motile spermatozoa pre-freezing and post-thawing, respectively, and a threshold of at least 83–90% morphologically normal spermatozoa depending on the specific deviation (personal communication, Geno SA).

For embryo production *in vivo*, we used 14 NR heifers in the age span of 12–15 months. In order to reduce individual differences and the maternal effect to a minimum, all animals came from the same genetic line with 28 years of targeted breeding, with high fertility and a low occurrence of clinical mastitis as target traits (Heringstad and Larsgard, 2010). They were free from disease or medical treatments according to their health records for the last 6 months before the sampling, they had no earlier inseminations and at least two visually registered estrus cycles. They were held indoors in the same free-range barn and received the same feeding throughout the study. Their body condition scorings were considered normal with an individual variation of 3.5–4.0, using a scale from 1-5 with increments of 0.25 (Edmonson et al., 1989), modified and adjusted for NR according to Gillund et al. (1999).

The ethical approval for the present study was provided by the Norwegian Food Safety authority with approval ID 11732. The combination of bull and heifer was randomized with block randomization; heifers were listed according to age, and every second animal was appointed a randomly chosen HF bull or LF bull, respectively. Randomization was performed using Sergeant, ESG, 2018, Epitools Epidemiological Calculators, Ausvet^2^. Semen from the two bulls with the lowest NRR were appointed to be used for two different heifers.

### Embryo Production and Collection

Embryo production was performed at The Animal Production Experimental Centre, NMBU in Ås, Norway in the spring of 2017. A protocol for synchronization and superovulation was developed for young NR heifers. The animals were synchronized with an intramuscular (i.m) injection of 2 ml cloprostenol 0.25 mg/ml (Estrumat vet., MSD Animal Health, Intervet International B.V., Nederland) twice at a 12-day interval and the following heat was visually detected. On day 9 after the first signs of standing heat, the 4-day administration with decreasing amounts of follicle stimulating hormone (FSH; Follitropin 500 IE and lutinizing hormone 500 IE, Pluset^®^ vet, Laboraotiros Calier, Barcelona, Spain) started.; Two i.m. injections were given daily (day one 2.0 ml, day two and three 1.5 ml and day four 1.0 ml). On the fourth day, a 2.0 ml i.m. injection of cloprostenol 0.25 mg/ml was administered in the morning and in the evening to induce luteolysis. The heat occurred after 2 days and AI was performed two or three times with 12 h in between depending on length of heat behavior.

Embryo flushing was performed 7 days after first AI. The ovaries were controlled for superovulation response using both manual palpation and rectal ultrasound, followed by an administration of epidural anesthesia. A Foley catheter was used to flush each uterine horn at least five times with ViGRO™ Complete Flush Solution (Vetoquinol, Lure Cedex, France. Previously Bioniche Animal Health, United States). The fluid was retrieved in an embryo collection filter (Emcon, Panningen, Netherland).

The embryos were transferred from the filter to a petri dish with SYNGRO ™ holding solution (Bioniche Animal Health, United States) and evaluated under a magnifying loupe at 40x magnification by three veterinarians according to the IETS guidelines (IETS-manual 3rd edition, IETS bovine *in vivo* embryo slide set tutorial, 2010). All embryos were then cleaned with phosphate buffered saline, moved to separate sterile mini tubes containing 1U/µl RNAsin in nuclease free water (RNasin Ribonuclease Inhibitor, Promega corporation, WI, United States), instantly frozen in liquid nitrogen, and stored in a −80°C freezer.

Embryos derived from HF sires were referred to as HF embryos, while those from the LF sires were referred to as LF embryos.

### RNA Extraction

Embryos with first grade quality and IETS stage 5 (early blastocyst), 6 (blastocyst) or 7 (expanded blastocyst) from both HF and LF groups were selected for further processing. RNA isolation was performed using the RNAqueous-Micro Total RNA isolation Kit (Thermo-Fisher Scientific, MA, United States) according to the producer’s instructions with some modifications; In order to break the zona pellucida and cell walls, embryos placed in 100 µl of the kit lysis solution were submitted to five cycles of 2 min freeze in liquid nitrogen and 2 min thaw in a 50°C water bath, followed by an incubation at 42°C overnight. The next day 50 µl of ethanol was added and isolation was continued according to the protocol. RNA was eluted twice in 6.0 and 6.5 µl elution solution, and the pooled eluate was treated with DNase1 as described by the producer. Production of cDNA was performed using the SMART-Seq v4 Ultra Low Input RNA Kit for sequencing (TaKaRa Bio Europe, Göteborg, Sweden) according to the producer’s protocol, using 10.5 µl of input RNA and 18 cycles of amplification. The Agencourt AMPure XP kit (Beckman Coulter, IN, United States) was used to purify the amplified cDNA as described in the Smart-Seq kit protocol. The cDNA concentration and quality were measured by Qubit fluorometer using the Qubit dsDNA HS Assay Kit (Thermo Fisher Scientific, MA, United States) and Agilent TapeStation D1000 using High Sensitivity reagents (Agilent, Santa Clara, CA, United States), respectively.

cDNA samples with concentrations between 0.262 ng/µl and 17.1 ng/µl (Supplementary Table S1) and sufficient quality according to the TapeStation profiles (Supplementary Figure S1) were selected for sequencing. In total, cDNA from 24 embryos were sent for sequencing at the Norwegian Sequencing Centre^3^, i.e., 13 embryos from four HF bulls and 11 embryos from three LF bulls. The total distribution between embryo stages 5, 6 and 7 were 1, 12 and 11 embryos, respectively. In the LF group, the embryo stages were 6 (*n* = 5) and 7 (*n* = 6), and in the HF group, the stages were 5 (*n* = 1), 6 (*n* = 7) and 7 (*n* = 5) (Supplementary Table S1).

### Sequencing and Data Analysis

Sequencing libraries from cDNA were prepared using SMARTer ThruPLEX DNA-prep kit (TaKaRa Bio United States Inc., San Jose, CA, United States) using unique indexes. Libraries were pooled and 150 bp paired end sequencing was performed on one lane of HiSeq 4,000 (Illumina, United States). Raw reads were processed using BBDuk (part of BBMap v34.56) (Bushnell, 2014) (parameters: ktrim = r k = 23 mink = 11 hdist = 1 tbo tpe qtrim = r trimq = 15 maq = 15 minlen = 36 forcetrimright = 149) to remove/trim low-quality reads and adapter sequences. Cleaned reads were aligned to the *Bos taurus* genome (ARS-UCD1.2; ENSEMBL release 95) using hisat2 v2.1.0 (Pertea et al., 2016) and the resulting sam files were converted to bam format using samtools v1.2. Reads mapping to the genes (ARS-UCD1.2; ENSEMBL release 95) were counted using featureCounts v1.4.6-p1 (Liao et al., 2014). Differential expression analysis was performed using DESeq2 v1.22.2 package (Love et al., 2014) in R v3. In brief, the counts were normalized followed by outlier detection (Cook’s distance), dispersion estimation (fittype: parametric) and statistical testing (hypothesis testing: Wald test). Independent filtering was performed which discarded 14,355 genes due to very low count values. Finally, multiple testing was performed using the Benjamini-Hochberg method. Genes with the adj.*p*-value less than 0.05 were considered to be significantly differentially expressed.

### Gene Set Enrichment Analysis

A gene set enrichment analysis of the DEGs was performed using the g:GOSt function in g:profiler version e104_eg51_p15_3922dba (Raudvere et al., 2019), which also integrated results from KEGG Pathway database, WikiPathways and Reactome. Separate analysis was performed for the genes highly expressed in the HF embryos and for the genes highly expressed in the LF embryos. A custom gene list consisting of 12,826 genes that were detected (adj.*p*-value not equal to “NA”) in the DE-analysis was used as background list for statistical domain scope (Supplementary Table S2). To correct for multiple testing, we used the Benjamini-Hochberg FDR algorithm and a threshold level of 0.05 for significance. For further functional analysis and visualization, we used the online version of Pathview v1.3.2^4^ (Luo et al., 2009; Luo and Brouwer, 2013; Luo et al., 2017).

The bovine genome is not as well studied and annotated as the human genome. To further enrich results interpretation, DEGs and the background list for statistical domain were converted to human ENSEMBL orthologs from bovine ENSEMBL IDs, by g:profiler and run though gene ontology analysis in g:profiler and pathway analysis in Pathviews, as described above.

## Results

### Output From Embryo Collection and Laboratory Work

All the heifers responded well to the superovulation protocol with normal size ovaries and no un-ovulated follicles. Sampling was normal in all heifers except for one, where collection from one of the uterine horns was not performed due to practical challenges. In total, 73 embryos were collected from 8 heifers inseminated with 6 LF bulls (=30 embryos) and 4 heifers inseminated with 4 HF bulls (=43 embryos), with an individual distribution of 1–19 embryos per animal. In the LF group, 17 of the 43 embryos collected had developed to the blastocyst stage, compared to 20 out of 30 in the HF group. From two heifers, no embryos could be found at collection. An overview of embryos sequenced from the different sires is given in Table 1. After the collection of embryos, Geno SA conducted an independent investigation of bulls that had previously been included in their breeding program, by analyzing genomic profiles, i.e., SNP data. That process revealed that one bull (K) had a deletion on chromosome 12.

**TABLE 1:** Bulls with high (HF) or low (LF) fertility, and number of embryos with sufficient material to sequence. NRR 56 = non return rate on day 56.

Bull fertility category	Bull ID	NRR 56	Number of embryos sequenced
HF	A	80.5	0
	B	79.7	0
	C	79.3	4
	D	79.3	2
	E	78.8	4
	F	78.7	3
LF	G	62.1	0
	H	61.9	8
	I	61.7	1
	J	56.8	0
	K	56.5	2
	L	49.3	0

### Output From Sequencing and Mapping

The sequencing resulted in an average of 12.5 million paired end reads per sample and more than 98% passed quality check. Read information and alignment statistics are provided in Supplementary Table S1. Raw sequence reads were uploaded to the NCBI SRA database with bioproject ID number: PRJNA762262.

The distribution of gene expression in the individual blastocysts was visualized by principal component analysis plots (Supplementary Figure S2). Two embryos (number 48 and 66) were shown as outliers and were removed from further analyses. These two samples also had an inferior cDNA quality according to the TapeStation profiles, which justified our decision. Due to this, the lowest cDNA concentration for samples analyzed in DESeq2 was 1.64 ng/µl. Embryos of IETS stages 5, 6 and 7 did not show strong signs of clustering within these groups in any of the three dimensions. The embryo of IETS stage 5 (from a HF bull) did not stand out as outlier or represent the highest or lowest value in any aspect of the PCA. Embryos from LF bulls compared to HF bulls clustered in the second dimension, but not in the first and third dimension.

### Differentially Expressed Genes

Among the 14,744 annotated genes that were detected during the analysis, there was a significant difference in the expression of 62 genes; 28 genes had a higher expression in the HF group, and 34 genes a higher expression in the LF group (Log2-foldchange > 0.64 adj.*p*-value < 0.05), see Table 2.

**TABLE 2:** Differentially expressed genes (mRNA) between embryos produced from low fertility (LF) and high fertility (HF) bulls. L = mean normalized count values in LF embryos. H = mean normalized count values in HF embryos.

ENSEMBL ID	Gene symbol	L	H	Log2 fold change	Adj.*p*-value	Functional description
Highly expressed in LF embryos						
ENSBTAG00000014046	*BPI*	573	0	10.90	1.72E-03	Bactericidal permeability increasing protein
ENSBTAG00000003305	*NCF1*	16	0	5.81	6.69E-03	Neutrophil cytosol factor 1
ENSBTAG00000047563	*CLDN9*	142	11	3.69	6.76E-03	Claudin 9
ENSBTAG00000026893	*EXOC3L4*	496	52	3.26	1.70E-03	*Bos taurus* exocyst complex component 3 like 4
ENSBTAG00000049434	Non-annotated gene	238	30	2.99	2.52E-03	
ENSBTAG00000051376	Non-annotated gene	33	6	2.43	2.52E-03	
ENSBTAG00000013854	*CALML5*	963	213	2.17	7.14E-10	Calmodulin like 5
ENSBTAG00000011839	*HMGCS1*	1949	471	2.05	6.13E-04	*Bos taurus* 3-hydroxy-3-methylglutaryl-CoA synthase 1
ENSBTAG00000054516	*CYP17A1*	293	76	1.95	2.60E-02	Steroid 17-alpha-hydroxylase/17,20 lyase
ENSBTAG00000017819	*PMVK*	310	93	1.74	1.21E-02	Phosphomevalonate kinase
ENSBTAG00000004905	*KRT19*	24,981	7,880	1.67	2.83E-02	Keratin 19
ENSBTAG00000003068	*MSMO1*	3,085	1,031	1.58	6.49E-04	Methylsterol monooxygenase 1
ENSBTAG00000006305	*AK1*	184	65	1.51	4.30E-03	*Bos taurus* adenylate kinase 1
ENSBTAG00000017864	*PRPH*	904	326	1.47	2.52E-03	*Bos taurus* peripherin
ENSBTAG00000055207	*SCD*	1,324	478	1.47	4.39E-03	*Bos taurus* stearoyl-CoA desaturase
ENSBTAG00000004075	*IDI1*	1,616	618	1.39	2.60E-04	*Bos taurus* isopentenyl-diphosphate delta isomerase 1
ENSBTAG00000012432	*FDFT1*	2,967	1,145	1.37	1.05E-02	*Bos taurus* farnesyl-diphosphate farnesyltransferase 1
ENSBTAG00000007840	*HMGCR*	1,178	465	1.34	4.30E-03	*Bos taurus* 3-hydroxy-3-methylglutaryl-CoA reductase
ENSBTAG00000004881	*MTHFD2*	1,085	440	1.30	2.84E-03	*Bos taurus* methylenetetrahydrofolate dehydrogenase (NADP + dependent) 2, methenyltetrahydrofolate cyclohydrolase
ENSBTAG00000014127	*PTGS2*	7,075	2,949	1.26	4.30E-03	*Bos taurus* prostaglandin-endoperoxide synthase 2
ENSBTAG00000055124	Non-annotated gene	53	23	1.23	2.76E-03	
ENSBTAG00000003948	*FDPS*	1,516	708	1.10	2.52E-03	Farnesyl diphosphate synthase
ENSBTAG00000003100	*SMTN*	209	100	1.05	2.76E-02	*Bos taurus* smoothelin
ENSBTAG00000004982	*GPLD1*	969	470	1.04	1.53E-02	*Bos taurus* glycosylphosphatidylinositol specific phospholipase D1
ENSBTAG00000032914	*SLC11A2*	1,023	513	1.00	2.51E-02	*Bos taurus* solute carrier family 11 member 2
ENSBTAG00000006471	*OSBPL11*	198	102	0.96	2.75E-02	Oxysterol binding protein like 11
ENSBTAG00000014227	*NDFIP2*	2,381	1,229	0.95	4.30E-03	*Bos taurus* Nedd4 family interacting protein 2
ENSBTAG00000019246	*SC5D*	605	319	0.92	4.56E-02	*Bos taurus* sterol-C5-desaturase
ENSBTAG00000055014	*SH3BGRL2*	2,429	1,352	0.85	3.07E-02	*Bos taurus SH3* domain binding glutamate rich protein like 2
ENSBTAG00000044015	*RBM12*	285	172	0.73	2.73E-02	*Bos taurus* RNA binding motif protein 12
ENSBTAG00000012317	*PNP*	3,762	2,302	0.71	4.78E-02	*Bos taurus* purine nucleoside phosphorylase
ENSBTAG00000016896	*HERPUD1*	531	327	0.70	2.52E-03	Homocysteine inducible ER protein with ubiquitin like domain 1
ENSBTAG00000017258	*ACSL3*	6,014	3,780	0.67	2.70E-02	*Bos taurus* acyl-CoA synthetase long chain family member 3
ENSBTAG00000011899	*USP4*	1,339	858	0.64	6.95E-03	Ubiquitin specific peptidase 4
**Highly expressed in HF embryos**						
ENSBTAG00000031825	Non-annotated gene	0	45	8.02	2.60E-04	
ENSBTAG00000046257	*GIMAP4*	1	68	5.85	3.57E-02	*Bos taurus* GTPase, *IMAP* family member 4
ENSBTAG00000014560	*HLX*	0	9	5.72	3.80E-02	*Bos taurus* H2.0 like homeobox
ENSBTAG00000030882	*hsd20b2*	7	81	3.48	1.85E-02	*Bos taurus* estradiol 17-beta-dehydrogenase 12-like (LOC508455)
ENSBTAG00000015836	Non-annotated gene	50	351	2.82	1.91E-04	
ENSBTAG00000010123	*APOE*	22	135	2.65	2.36E-02	Apolipoprotein E
ENSBTAG00000014596	*EFHD1*	153	823	2.43	1.70E-03	EF-hand domain family member D1
ENSBTAG00000027444	*SVIL*	12	60	2.37	4.98E-02	*Bos taurus* supervillin
ENSBTAG00000054434	Non-annotated gene	6	25	2.08	3.33E-02	
ENSBTAG00000033429	*FAM229B*	7	26	1.96	4.92E-02	*Bos taurus* family with sequence similarity 229 member B
ENSBTAG00000049950	Non-annotated gene	9	32	1.86	4.92E-02	
ENSBTAG00000026758	Non-annotated gene	22	77	1.83	2.60E-02	
ENSBTAG00000017094	*SHMT1*	63	209	1.72	2.73E-02	Serine hydroxymethyltransferase 1
ENSBTAG00000038384	*KRT5*	434	1,210	1.48	1.21E-02	Keratin 5
ENSBTAG00000054234	Non-annotated gene	228	632	1.47	2.86E-02	
ENSBTAG00000003568	*CLDN10*	417	1,120	1.43	4.44E-03	*Bos taurus* claudin 10, transcript variant 2
ENSBTAG00000004386	*SOCS1*	105	274	1.39	4.30E-03	Suppressor of cytokine signaling 1
ENSBTAG00000012511	*BAD*	306	787	1.36	1.72E-03	*Bos taurus BCL2* associated agonist of cell death
ENSBTAG00000003043	*GNG2*	148	373	1.33	2.09E-02	G protein subunit gamma 2
ENSBTAG00000006086	*MMP28*	110	278	1.33	1.72E-03	Matrix metallopeptidase 28
ENSBTAG00000022028	*DERL3*	114	257	1.17	2.52E-03	*Bos taurus* derlin 3
ENSBTAG00000013922	*MOSPD1*	196	435	1.15	4.47E-02	*Bos taurus* motile sperm domain containing 1
ENSBTAG00000020528	*PCOLCE*	126	273	1.12	1.01E-02	*Bos taurus* procollagen C-endopeptidase enhancer
ENSBTAG00000003222	*ASNS*	287	574	1.00	4.18E-02	Asparagine synthetase (glutamine-hydrolyzing)
ENSBTAG00000010740	*CLTB*	2,843	5,223	0.88	4.21E-02	Clathrin light chain B
ENSBTAG00000052249	Non-annotated gene	41	71	0.80	2.60E-02	
ENSBTAG00000021111	*POU5F1*	10,804	18,443	0.77	2.26E-03	*Bos taurus POU* class 5 homeobox 1
ENSBTAG00000017932	*CCDC84*	183	286	0.65	3.75E-02	*Bos taurus* coiled-coil domain containing 84

An overview over the significant pathways and gene ontology (GO)-terms found in g:profiler is provided in Table 3. The full DE-list revealed three significantly affected pathways in Pathview, represented by 9 different genes; terpenoid backbone synthesis (4 genes represented), insulin signaling pathway (3 genes represented) and leukocyte transendothelial migration (2 genes represented).

**TABLE 3:** Significant pathways and gene ontology terms found in g:profiler for the differentially expressed genes between LF embryos and HF embryos.

Pathway or gene ontology term	Pathway ID	Adj. *p*-value	Genes represented
**Highly expressed in LF embryos**			
sterol biosynthetic process	GO:0016126	1.51E-08	*HMGCS1, PMVK, MSMO1, IDI1, FDFT1, HMGCR, SC5D*
cholesterol biosynthetic process	GO:0006695	1.42E-06	*HMGCS1, PMVK, IDI1, FDFT1, HMGCR, SC5D*
secondary alcohol biosynthetic process	GO:1902653	1.42E-06	*HMGCS1, PMVK, IDI1, FDFT1, HMGCR, SC5D*
steroid biosynthetic process	GO:0006694	8.33E-06	*HMGCS1, PMVK, MSMO1, IDI1, FDFT1, HMGCR, SC5D*
sterol metabolic process	GO:0016125	9.39E-06	*HMGCS1, PMVK, MSMO1, IDI1, FDFT1, HMGCR, SC5D*
lipid biosynthetic process	GO:0008610	3.63E-05	*HMGCS1, PMVK, MSMO1, SCD, IDI1, FDFT1, HMGCR, PTGS2, FDPS, SC5D, ACSL3*
isoprenoid biosynthetic process	GO:0008299	5.45E-05	*HMGCS1, PMVK, IDI1, HMGCR, FDPS*
isoprenoid metabolic process	GO:0006720	6.19E-05	*HMGCS1, PMVK, IDI1, FDFT1, HMGCR, FDPS*
organic hydroxy compound biosynthetic process	GO:1901617	2,59E-04	*HMGCS1, PMVK, MSMO1, IDI1, FDFT1, HMGCR, SC5D*
cholesterol metabolic process	GO:0008203	4,00E-04	*HMGCS1, PMVK, IDI1, FDFT1, HMGCR, SC5D*
secondary alcohol metabolic process	GO:1902652	5,45E-04	*HMGCS1, PMVK, IDI1, FDFT1, HMGCR, SC5D*
alcohol biosynthetic process	GO:0046165	8,84E-04	*HMGCS1, PMVK, IDI1, FDFT1, HMGCR, SC5D*
steroid metabolic process	GO:0008202	9,62E-04	*HMGCS1, PMVK, MSMO1, IDI1, FDFT1, HMGCR, SC5D*
lipid metabolic process	GO:0006629	5,31E-03	*HMGCS1, PMVK, MSMO1, SCD, IDI1, FDFT1, HMGCR, PTGS2, FDPS, GPLD1, SC5D, ACSL3*
small molecule metabolic process	GO:0044281	2,17E-02	*HMGCS1, PMVK, AK1, SCD, IDI1, FDFT1, HMGCR, MTHFD2, PTGS2,* Non-annotated gene: ENSBTAG00000014127, *GNG2, SC5D, PNP, ACSL3*
cellular lipid metabolic process	GO:0044255	4,60E-02	*HMGCS1, PMVK, SCD, IDI1, FDFT1, HMGCR, PTGS2, FDPS, GPLD1, ACSL3*
small molecule biosynthetic process	GO:0044283	4,94E-02	*HMGCS1, PMVK, SCD, IDI1, FDFT1, HMGCR, PTGS2, SC5D*
Metabolic pathways	KEGG:01100	2,91E-08	*HMGCS1,* Non-annotated gene: ENSBTAG00000054516, *PMVK, MSM O 1, AK1, SCD, IDI1, FDFT1, HMGCR, MTHFD2, PTGS2*, Non-annotated gene: ENSBTAG00000014127, *GNG2, FDPS, GPLD1, SC5D, PNP, ACSL3*
Terpenoid backbone biosynthesis	KEGG:00900	8,78E-08	*HMGCS1, PMVK, IDI1, HMGCR, FDPS*
Steroid biosynthesis	KEGG:00100	4,59E-04	*MSMO1, FDFT1, SC5D*
PPAR signaling pathway	KEGG:03320	1,10E-02	*HMGCS1, SCD, ACSL3*
Cholesterol biosynthesis	REAC:R-BTA-191273	3,13E-08	*MSMO1, IDI1, FDFT1, HMGCR, SC5D*
Metabolism of steroids	REAC:R-BTA-8957322	2,02E-05	*MSMO1, IDI1, FDFT1, HMGCR, SC5D*
Metabolism of lipids	REAC:R-BTA-556833	8,38E-04	*MSMO1, IDI1, FDFT1, HMGCR, PTGS2, SC5D, ACSL3*
Metabolism	REAC:R-BTA-1430728	1,72E-02	*MSM O 1, IDI1, FDFT1, HMGCR, MTHFD2, PTGS2, SC5D, PNP, ACSL3*
Cholesterol Biosynthesis	WP:WP1070	2,61E-16	*HMGCS1, PMVK, MSMO1, IDI1, FDFT1, HMGCR, FDPS, SC5D*
SREBP signalling	WP:WP3194	1,25E-05	*HMGCS1, IDI1, FDFT1, HMGCR, FDPS*
SREBF and miR33 in cholesterol and lipid homeostasis	WP:WP3137	2,14E-02	*HMGCS1, HMGCR*
Statin Pathway	WP:WP1041	4,16E-02	*FDFT1, HMGCR*
**Highly expressed in HF embryos**			
G protein complex (CACNA1A, GNB1, GNG2)	CORUM:3216	4,99E-02	*GNG2*

Genes that were highly expressed in the LF group (*n* = 34) were associated with 29 GO-terms for biological processes and pathways, most prominently biosynthetic process- and metabolism of sterol, steroids, isoprenoids and cholesterol. Pathway analysis in KEGG, Reactome and WikiPathways through g:profiler showed enrichment of terpenoid backbone synthesis, and metabolism and biosynthesis of cholesterol, steroids and lipids. The individual transcript showing the biggest difference between the groups, with higher expression in the LF group, was mRNA coding for bactericidal permeability increasing protein (gene symbol *BPI*, log2fold change = 10.897).Another transcript, with higher expression in the LF group was mRNA coding for neutrophil cytosol factor 1 (gene symbol *NCF1*, log2 fold change = 5.812), a protein engaged in leukocyte transendothelial migration and a NADPH oxidase regulator. The leukocyte transendothelial migration pathway was further represented by the gene claudin 9 (*CDLN9*, log2 fold change = 3.688), the third most up-regulated transcript in the LF group and coding for cell adhesion molecules (CAMs).

Genes that were highly expressed in the HF group (*n* = 28) were associated with only one GO-term through Corum in g:profiler, the G protein complex. The biggest foldchange was represented by a non-annotated gene (ENSBTAG00000031825, log2fold change = 8.016) followed by *GIMAP4* (log2fold change = 5.850) and *HLX* (log2fold change = 5.724).

### Non-Annotated Genes

Out of all significant DEGs in the DE-list, nine genes were described as non-annotated genes, of which seven did not have any pathway and GO-terms associated. Three of the non-annotated genes were highly expressed in LF embryos, and six in HF embryos (Table 2). Some of these represented a very high level of difference between the LF and HF groups, with the highest log2fold change of 8.016 and 2.823 in the HF group, and 2.994 and 2.425 in the LF group.

### Human Orthologs

To enrich results interpretation, we used human orthologs, as the human genome is better studied and annotated. Out of the 62 bovine genes from the DE-list, g:profiler found 58 human orthologues (Supplementary Table S3). Only four genes did not have a human orthologue. The Pathview program pathways represented by these orthologues matched the pathways for the bovine genome. Out of the 29 GO-terms or pathways linked to genes highly expressed in the LF group, 27 were enriched in the human orthologs. Out of these 27, 16 terms or pathways were each associated with one or more genes in the human orthologs compared to the original material. In 6 other enriched processes or pathways, the same number of genes represented both the cattle and human ortholog outcome. Fourteen terms or pathways that did not appear in the original material, were shown in the analysis of orthologs. These pathways were strongly related to, or represented a higher hierarchy of, the same pathways that had already been identified. Only two pathways in the human orthologs, Omega-9 FA synthesis and Cuteneous photosensitivity, were not related but these were only represented by two and three genes, respectivley. For the genes that were highly expressed in the HF group, the analysis of human orthologs did not give any GO-terms or pathways.

## Discussion

The present study is the first to compare the complete gene expression of *in vivo* produced embryos from sires with high and low field fertility, measured as high or low NRR respectively. Gene expression differed significantly between the two groups, and we identified several pathways affected by the field fertility of the bull. There was consensus between the different databases used by g:profiler (KEGG, Reactome, Corum and Wikipathways). Our findings were further strengthened by analysis of human orthologues, which were related to almost identical pathways.

We collected 30 embryos from four high fertility bulls and 43 embryos from six low fertility bulls, with individual differences of 1–19 embryos per bull. The deletion found in one LF embryo leads to embryonic or fetal death in homozygous conceptuses, and was described by Kadri et al. (2014). The embryos from LF sires showed a tendency of greater variation in developmental stage, where only 39.5% (17/43) had developed to the blastocyst stage, compared to 66.6% (20/30) in the HF group. Some earlier studies support the positive relationship between embryo cleavage or blastocyst rate *in vitro*, and field fertility in the bull (Zhang et al., 1997; Ward et al., 2001; O’Callaghan et al., 2021), while others do not (Kropp et al., 2017).

We identified 62 genes differentially expressed between embryos produced from low fertility and high fertility bulls. This seemingly low number is comparable to the findings in two similar studies of male contribution to embryo development. Both studies used RNA-sequencing of *in vitro* produced embryos, identifying 65 differentially expressed genes for the blastocyst stage and the 2-4 cell stage embryos, respectively (Kropp et al., 2017; Gross et al., 2019). Another study, that compared morphologically degenerative embryos on day 8 to normally developed blastocysts, found 47 differentially expressed genes (Huang and Khatib, 2010), all suggesting that a change in only a slight number of transcripts can be responsible for gross changes in the embryo.

### Enriched Pathways and Gene Ontology Terms

Embryos derived from LF bulls showed a higher genetic expression corresponding to a more active metabolism. These results are in correlation with earlier literature, proposing an association between a high survival rate in the embryo, and a lower level of metabolism (Leese, 2002; Baumann et al., 2007; Leese et al., 2007). One of the pathways that was highly expressed in the LF group was the terpenoid backbone biosynthesis (Kanehisa and Goto, 2000; Kanehisa, 2019; Kanehisa et al., 2021) (Supplementary Figure S3), which initiates the production of sterol isoprenoids, such as cholesterol, and non-sterol isoprenoids (Buhaescu and Izzedine, 2007; Miziorko, 2011). The products derived from this pathway play an essential role in various cellular processes such as cell growth and differentiation, and cell signaling (Goldstein and Brown, 1990). One of the continuations of this pathway; steroid biosynthesis (Kanehisa and Goto, 2000; Kanehisa, 2019; Kanehisa et al., 2021) (Supplementary Figure S4) was also highly expressed in the LF group. The increased activity in these connected pathways was represented by nine transcripts in the present study. The first three, *HMGCS1*, *HMGCR* and *PMVK* all code for enzymes in the production of mevalonate. HMGCR has been described as a rate limiting enzyme and is of major importance for the entire downstream process (Goldstein and Brown, 1990). Both HMGCR and HMGCS1 underlie well-studied, vast mechanisms of regulation. One of those mechanisms is an end-product feedback system that allows any absence of sterol isoprenoids to activate the transcription of the *HMGCR* gene through a family of transcription factors called sterol regulatory binding proteins (SREBP) (Brown and Goldstein, 1997; Buhaescu and Izzedine, 2007).

The next two genes represented in these pathways, *DID1* and *FDPS*, encode enzymes that catalyze the further descendance of metabolites towards steroid biosynthesis while four other genes, *FDFT1*, *MSMO1*, *SC5DL* and *CYP17A1* encode enzymes that lead the metabolism down to the biosynthesis of cholesterol (and several other steroids) and steroid hormones. One study demonstrated that an increased SREBP activity not only acted on *HMGCR* and *HMGCS1*, but also increased the mRNA expression of several enzymes along the entire pathway of cholesterol production (Sakakura et al., 2001). Based on this information, we speculate that the high expression of the mentioned enzymes in the LF embryos, could be a result of any dysfunction of the pathways related to sterols, or any exaggerated degradation or demands of its products. Cholesterol is essential for the developing embryo, as it forms part of the cell membrane, and acts in cell signaling crucial for developmental patterning, in collaboration with the hedgehog gene family (Porter et al., 1996; Roux et al., 2000). It can be toxic in too large quantities and its production entails high metabolic costs for cells to produce. Hence, its production is under strict regulation (Sharpe and Brown, 2013). The complete knockout of the *HMGCR* gene in mouse embryos resulted in the recovery of morphologically normal blastocyst but no later developmental stages. This suggest that at least some of HMGCR products are essential for development from the blastocyst stage, either prior to implantation, or for the implantation process itself (Ohashi et al., 2003). Equally, *CYP17A1* disruption leads to early embryonic lethargy in murine embryos (Bair and Mellon, 2004). Hence, a suboptimal level of cholesterol and/or its precursors in the LF embryos would lower their developmental potential. This marks a difference between LF and HF embryos that could explain at least part of the reason for low field fertility in the LF bulls.

Interestingly, the sterol biosynthetic process and cholesterol pathway have been highlighted in the comparison between morphologically similar *in vitro* and *in vivo* produced embryos (Driver et al., 2012). *In vitro* embryos have a reduced developmental potential from the zygote to blastocyst stage, and a lower success in embryo transfer (Rizos et al., 2008). Driver et al. (2012) performed a transcriptome study in stage 7 blastocysts, where the *in vitro* group had an increased expression in 11 genes related to the cholesterol pathway. These genes included *HMGCS1*, *HMGCR*, *PMVK*, *IDI1* and *FDFT1* which are all identical to our findings. The present study only analyzed *in vivo* produced embryos, but equal to the study by Driver et al. (2012), it compared the transcripts of embryos with a hypothetical difference in developmental potential. The fact that the results of the two studies are in agreement, confirms the central role of the mentioned pathways for successful embryo development.

The leukocyte transendothelial migration pathway (Kanehisa and Goto, 2000; Kanehisa, 2019; Kanehisa et al., 2021) (Supplementary Figure S5) was represented by two of the most highly expressed genes in the LF group. Interestingly, the pathway was also represented by one gene with a high expression in the HF group. Earlier literature pointed to several similarities between leukocyte transendothelial migration and human implantation, stating that both processes use the same mechanisms of adhesion, molecular interaction and migration (Genbacev et al., 2003; Dominguez et al., 2005). Hence, one explanation for the low bull fertility, might be through an effect on the control of implantation. The leukocyte transendothelial migration pathway was represented by *NCF1* and *CLDN9* in the LF group, and *CLDN10* in the HF group. Claudins (CLDN9 and CLDN10) also have a role in embryo development, independent of their role in this pathway. The claudin gene family forms part of tight junctions, which are transmembrane compounds with functions in the maintenance of apical-basal polarity and cell adhesion (Gupta and Ryan, 2010). Tight junctions are crucial for morphogenesis (Furuse and Moriwaki, 2009), and a loss of function-study revealed that some claudins are essential for the formation of the murine blastocyst (Moriwaki et al., 2007). In a review of claudin function in embryogenesis, the authors hypothesized that the combined expression of claudin, or the “claudin signature,” is critical to embryonic tissues (Gupta and Ryan, 2010). The specific importance of a high expression of *NCF1* is uncertain. NCF1, also known as p47phox, takes part in the production of reactive oxygen species (ROS) through its role in NADPH oxidase (Babior, 2004). The change in expression of *NCF1* might point to a change in the redox state (reviewed by Harvey et al., 2002) in any of the two embryo groups. Changes in the embryo redox state through the limited accumulation of ROS is naturally occurring, enabling developmental progress in the embryo (Dennery, 2007). However, it also controls programmed cell death (Pierce et al., 1991), and in excess, oxidative stress is embryotoxic (Dennery, 2007). Consequently, one possible causative factor of the poorer outcome for the LF embryos could be through a lower competence in the regulation of redox activity. This hypothesis is supported by the findings in one earlier study of the sire’s contribution to embryo development (Kropp et al., 2017).

Another pathway differing between the HF and LF groups was the insulin signaling pathway (Kanehisa and Goto, 2000; Kanehisa, 2019; Kanehisa et al., 2021) (Supplementary Figure S6). Two genes were abundant in the HF group: *SOCS1* and *BAD*. *SOCS1* encodes an enzyme which inhibits the action of the insulin receptor (Mooney et al., 2001), hence suppresses the full signaling pathway, which contains PI3K-Akt signaling. PI3K-Akt signaling has a central role in embryo survival, regulating differentiation and cell growth, proliferation, anti-apoptosis and calcium metabolism (Leese and Brison, 2015, p.184). The enzyme encoded by *SOCS1* also has several roles in the negative feedback mechanism of cytokine signaling (Krebs and Hilton, 2001). Cytokines are produced by the embryo itself, as well as the female reproductive tracts as a mediator of maternal-embryo communication. They affect a vast range of processes, again related to cell differentiation and cell survival (Leese and Brison, 2015, p.173–174). *SOCS1* expression is a product of both interferon-γ and interleukin-4 and the protein encoded by *SOCS1* has a negative feedback on these cytokines (Fujimoto and Naka, 2003). Interferon-γ has several important roles in embryo development, but excess production is detrimental (Leese and Brison, 2015, p.193), implying that a well-functioning regulatory mechanism is beneficial for the embryo. *BAD*, on the other hand, encodes an enzyme that is inhibited by the activation of the insulin signaling pathway, so a higher expression could be a result of the elevated activity in SOCS1. BAD is an antagonist of apoptosis, which is interesting since a higher apoptotic cell ratio indicates a lower developmental competence in the embryo (Maddox-Hyttel et al., 2003). Certainly, the lower degree of apoptosis in the HF group would make a logical explanation for a higher developmental potential. The apoptotic cell ratio is inversely correlated to early cleavage in zygotes (Byrne et al., 1999), which is again positively correlated to bull fertility (Ward et al., 2001). However, one study intended to demonstrate a direct association between bull fertility and apoptotic cell ratio, but failed to do so (Vandaele et al., 2006).

### Other Transcripts

Some of the of the most highly expressed genes in the two groups were not represented in any pathways. *GIMAP4*, encoding a small GTPase active in the immune system (Heinonen et al., 2015) was highly expressed in the HF embryos. It regulates cytokine secretion in the early human CD4^+^ Th lymphocytes and initiates the secretion of interferon-γ (Heinonen et al., 2015). GIMAP4 is also an important regulator of calcium signaling (Schnell et al., 2006), a process which in recent years has been shown to have several functions in the pre- and peri implantation period (Leese and Brison, 2015, p.158–164). To the authors knowledge, the exact role of *GIMAP4* in embryo development has not yet been defined. *HLX* was another highly expressed gene in the HF embryos. Similar to SOCS1, HLX is also a regulator of cytokines, allowing trophoblast proliferation and the development of the placenta (Rajaraman et al., 2010). This, again, proposes an association between the paternal contribution and the mechanism for implantation in our material.

Another interesting finding in the HF group was the higher expression of *POUF51.* This gene encodes the transcription factor Oct4, which is essential for pluripotency and the formation of an intracellular matrix (Nichols et al., 1998). Our results could denote that HF embryos are more competent in this matter, and are more likely to develop beyond the blastocyst stage. The highest DE seen in the HF group was of a non-annotated gene; ENSBTAG00000031825. Its homologue *C19orf12* (e value 0.0) has recently been shown to be important in neuronal development in zebrafish embryos, as a downregulation of the gene had severe effects on brain morphology and resulted in embryo death before day 7. Its function was suggested to be related to lipid metabolism even though the cellular mechanism is poorly understood (Mignani et al., 2020). The higher expression of this gene in our HF embryos is indeed an interesting finding that could explain differences in bull fertility but requires more investigation.

*BPI* was the single gene showing the highest DE in the LF group. The gene product is a lipid-transfer protein with the capacity to neutralize endotoxin. In humans, it is produced by neutrophils and the epithelial lining of mucosa as part of an antimicrobial defense mechanism (Schultz and Weiss, 2007). Proteins encoded by *BPI* and the *BPI*-like PLUNC genes from the same superfamily, have been found in the seminal plasma of rams (Soleilhavoup et al., 2014; van Tilburg et al., 2020), and the spermatozoa membrane of mice (Zhou et al., 2014) and rodents (Yano et al., 2010), and are hypothesized to have a role in the sperm-oocyte fusion process (Li et al., 2013). Even if BPI were to be identified in the semen of bulls, the finding in the embryos of the present study is likely not a direct result of paternal transcripts deposited to the oocyte at fertilization, since these transcripts start to degrade at EGA and should not be abundant in the analyses of embryonic gene expression at the blastocysts stage (Graf et al., 2014; Jiang et al., 2014). Neither *BPI* in cattle, nor its human orthologue has been assigned to any pathway, and to the authors knowledge, the role of *BPI* in embryo development has not been reported in earlier studies. However, one RNA-seq study that compared different stages of *in vivo* produced cow embryos found that *BPIAF1* (a *BPI*-like PLUNC gene) is a hub gene in blastocysts. This information was validated with literature of human and mice blastocysts (Jiang et al., 2014). Further research is necessary to study the role of *BPI* in embryo development.

### Limitations of the Study

Defining the significance of our interpretations to our findings is challenging, given that cell signaling in embryo development is controlled by a vast number of processes with overlapping actions and shared receptors (Leese and Brison, 2015, p.180). Superovulation could have altered the gene expression of some genes in the current study compared to a normal *in vivo* produced embryo (Mundim et al., 2009), but this alteration applies to both LF and HF embryos and should not affect the differences between the groups. Moreover, it is not certain that the death of the conceptuses from LF bulls occurs at the blastocyst stage or before implantation, even if most embryo death probably occurs before day 8 after conception (Diskin et al., 2016). Equally, although fertilization failure is not the main problem of non-successful coupling (Sreenan and Diskin, 1986; Diskin et al., 2016), we cannot rule out that the LF bulls in our study might have had a weak fertilization capacity. However, two recent studies on early embryo development in high and low fertility bulls, showed no difference in fertilization rate (Kropp et al., 2017; O'Callaghan et al., 2021), while one showed a difference in the development until day 7 between the two groups (O’Callaghan et al., 2021). In the present study, it is uncertain whether embryos were lost in the *in vivo* collection process, or whether the embryos that did not develop to the blastocyst stage, had the potential to do so. Equally, although the maternal effect was reduced by using very similar heifers with equal living conditions, each embryo was inevitably affected to some extent by the individual differences in the genetics of the heifers. Another limitation of the study was that we had to choose a mix of embryos of IETS stages 5, 6 and 7, which could have affected the relative expression of some genes. However, despite of this and individual differences, the distribution in the PCA plots supports the argument that the selected embryo stages are sufficiently uniform to study the differences between the HF and LF bulls. Equally, it would have been interesting to include embryos of all qualities in the two groups, and not only the highest quality. However, it is well known that gene expression varies between individuals and if this variation is too large it may obscure potential differences between groups of individuals. Embryos of different quality are expected to differ in expression profiles. Hence, in order to keep the individual variation within the groups to a minimum, only embryos of the highest quality were used.

### Significance and Future Perspective

To our knowledge, this is the first study to investigate the transcriptome of *in vivo* produced embryos for the influence of paternal field fertility. Comparing our results to a similar study that investigated bull field fertility and embryo transcriptomic profiles in *in vitro* produced blastocysts (Kropp et al., 2017), we found few evident similarities in the genes or pathways that were differentially expressed. This underlines the importance of studying *in vivo* produced embryos even though it is a challenging approach. To understand the sire’s effect on the embryo, one needs to study a complex relationship between several factors such as aspects of the semen and spermatozoa, molecular genetics and epigenetics. The bull’s effect on the embryo as reported in the present study, might be caused by either bull DNA, or regulations by proteins, transcriptome or epigenetic factors deposited in the oocyte at fertilization. Regardless of the type of contribution, it is certain that it originates from the spermatozoa. Therefore, further epigenetic investigations of both spermatozoa and the resulting embryos from the same bulls would be highly interesting.

The present study adds important information to the current understanding of the paternal influence on the genetic components in embryo development. Although the field of bull fertility has received clear attention and progress during the last decade, further research is needed to clarify this complex matter, with the goal to find biomarkers that aid the prediction of bull fertility.

## Conclusion

There was a tendency of a higher blastocyst recovery rate from heifers inseminated with the HF bulls compared to the LF bulls. Sires with a high or low field fertility produced embryos with different transcriptomic profiles, represented by the expression of 62 transcripts, several of them known to be crucial for embryo survival and development potential. The LF embryos showed a higher activity in pathways related to sterol metabolism and terpenoid backbone synthesis, while HF embryos expressed genes linked to anti-apoptosis and the regulation of cytokine signaling. The leukocyte transendothelial migration and the insulin signaling pathways were associated with enrichments in both groups. Our results suggest that the sire’s genetic contribution affects all these important processes, linked to pre-and peri implantation regulation in the developing embryo. The mechanism or contributing component in the spermatozoa that affects the embryo demand further investigation.

## Data Availability

The original contributions presented in the study are publicly available. This data can be found here:PRJNA762262.

## Footnotes

1
www.geno.no.

2
http://epitools.ausvet.com.au.

3
www.sequencing.uio.no.

4
https://pathview.uncc.edu/.
