# Supplementary material for: Gene Expression in Embryos From Norwegian Red Bulls With High or Low Non Return Rate: An RNA-Seq Study of in vivo-Produced Single Embryos
Source: Front Genet. 2022 Jan 14;12:780113. doi: 10.3389/fgene.2021.780113 (PMC8795813; doi:10.3389/fgene.2021.780113)
Supplement: Supplementary file 1 [file DataSheet2.PDF]

Supplementary Material

Supplementary Table 1: Outcome from cDNA production and RNA-sequencing

Fertility group	Embryo ID_Bull ID	IETS stage	cDNA concentration (ng/µl)	Raw read pairs	Clean read pairs	Clean reads %	Overall alignment % (clean reads)	Assigned fragments (read pairs)
HF	6HF_F	6	1.92	14 452 521	14 272 329	98.75	94.14	12 035 422
	7HF_F	6	4.26	10 576 857	10 372 368	98.07	94.46	8 875 618
	9HF_F	7	2.10	10 727 374	10 570 662	98.54	94.77	9 053 036
	22HF_E	7	8.34	11 151 012	11 013 848	98.77	95.28	9 255 163
	23HF_E	7	1.79	11 220 469	11 055 601	98.53	90.38	8 489 011
	25HF_E	6	7.72	13 618 537	13 410 107	98.47	94.96	11 458 301
	27HF_E	6	2.68	12 007 766	11 859 178	98.76	95.02	10 080 841
	29HF_C	7	5.64	10 680 818	10 530 598	98.59	95.47	9 082 237
	31HF_C	6	5.78	10 824 828	10 663 433	98.51	94.96	9 113 239
	32HF_C	6	3.92	14 597 117	14 357 428	98.36	93.46	12 042 081
	35HF_C	5	17.1	13 272 288	13 093 999	98.66	95.58	11 396 884
	41HF_D	7	6.00	12 660 892	12 498 892	98.72	93.20	10 191 932
	42HF_D	6	4.86	11 989 521	11 780 644	98.26	94.26	9 871 632
LF	13LF_K	6	1.64	13 678 471	13 487 721	98.61	94.38	11 234 348
	14LF_K	6	2.00	12 423 286	12 245 907	98.57	94.66	10 409 460
	48LF_H*	6	0.926	13 898 832	13 648 931	98.20	80.94	8 527 018
	49LF_H	7	6.72	21 583 312	21 275 878	98.58	94.98	17 867 228
	50LF_H	7	8.40	9 165 231	8 995 376	98.15	93.84	7 504 194
	51LF_H	7	7.46	12 364 514	12 185 164	98.55	95.38	10 522 277
	52LF_H	7	9.14	10 978 344	10 798 704	98.36	95.27	9 303 217
	53LF_H	7	12.2	11 824 901	11 674 310	98.73	95.25	10 043 333
	57LF_H	7	7.04	11 433 681	11 245 667	98.36	94.14	9 455 553
	63LF_H	6	9.12	13 409 146	13 245 803	98.78	95.20	11 418 022
	66LF_I*	6	0.262	13 679 142	13 494 347	98.65	91.41	10 343 817

**These samples were considered outliers due to poor cDNA concentration and sequencing quality, and hence removed from further analyses.*

Supplementary Table 3: Human orthologues

Bovine gene ID	Gene symbol	Log2 foldchange	Adj.p-value	Human orthologue
		**Highly expressed in LF embryos**		
ENSBTAG00000014046	*BPI*	10.90	1.72E-03	ENSG00000101425
ENSBTAG00000003305	*NCF1*	5.81	6.69E-03	ENSG00000158517
ENSBTAG00000047563	*CLDN9*	3.69	6.76E-03	ENSG00000213937
ENSBTAG00000026893	*EXOC3L4*	3.26	1.70E-03	ENSG00000205436
ENSBTAG00000049434	(Non-annotated gene)	2.99	2.52E-03	ENSG00000155380
ENSBTAG00000051376	(Non-annotated gene)	2.43	2.52E-03	ENSG00000151729
ENSBTAG00000013854	*CALML5*	2.17	7.14E-10	ENSG00000178372
ENSBTAG00000011839	*HMGCS1*	2.05	6.13E-04	ENSG00000112972
ENSBTAG00000054516	*CYP17A1*	1.95	2.60E-02	ENSG00000148795
ENSBTAG00000017819	*PMVK*	1.74	1.21E-02	ENSG00000163344
ENSBTAG00000004905	*KRT19*	1.67	2.83E-02	ENSG00000171345
ENSBTAG00000003068	*MSMO1*	1.58	6.49E-04	ENSG00000052802
ENSBTAG00000006305	*AK1*	1.51	4.30E-03	ENSG00000106992
ENSBTAG00000017864	*PRPH*	1.47	2.52E-03	ENSG00000135406
ENSBTAG00000055207	*SCD*	1.47	4.39E-03	ENSG00000099194
ENSBTAG00000004075	*IDI1*	1.39	2.60E-04	ENSG00000067064
ENSBTAG00000012432	*FDFT1*	1.37	1.05E-02	ENSG00000079459
ENSBTAG00000007840	*HMGCR*	1.34	4.30E-03	ENSG00000113161
ENSBTAG00000004881	*MTHFD2*	1.30	2.84E-03	ENSG00000065911
ENSBTAG00000014127	*PTGS2*	1.26	4.30E-03	ENSG00000073756
ENSBTAG00000055124	(Non-annotated gene)	1.23	2.76E-03	No orthologue
ENSBTAG00000003948	*FDPS*	1.10	2.52E-03	ENSG00000160752
ENSBTAG00000003100	*SMTN*	1.05	2.76E-02	ENSG00000183963
ENSBTAG00000004982	*GPLD1*	1.04	1.53E-02	ENSG00000112293
ENSBTAG00000032914	*SLC11A2*	1.00	2.51E-02	ENSG00000110911
ENSBTAG00000006471	*OSBPL11*	0.96	2.75E-02	ENSG00000144909
ENSBTAG00000014227	*NDFIP2*	0.95	4.30E-03	ENSG00000102471
ENSBTAG00000019246	*SC5D*	0.92	4.56E-02	ENSG00000109929
ENSBTAG00000055014	*SH3BGRL2*	0.85	3.07E-02	ENSG00000198478
ENSBTAG00000044015	*RBM12*	0.73	2.73E-02	ENSG00000244462
ENSBTAG00000012317	*PNP*	0.71	4.78E-02	ENSG00000198805
ENSBTAG00000016896	*HERPUD1*	0.70	2.52E-03	ENSG00000051108
ENSBTAG00000017258	*ACSL3*	0.67	2.70E-02	ENSG00000123983
ENSBTAG00000011899	*USP4*	0.64	6.95E-03	ENSG00000114316

Highly expressed in HF embryos				
ENSBTAG00000031825	(Non-annotated gene)	8.02	2.60E-04	ENSG00000131943
ENSBTAG00000046257	*GIMAP4*	5.85	3.57E-02	ENSG00000133574
ENSBTAG00000014560	*HLX*	5.72	3.80E-02	ENSG00000136630
ENSBTAG00000030882	*hsd20b2*	3.48	1.85E-02	No orthologue
ENSBTAG00000015836	(Non-annotated gene)	2.82	1.91E-04	ENSG00000283632
ENSBTAG00000010123	*APOE*	2.65	2.36E-02	ENSG00000130203
ENSBTAG00000014596	*EFHD1*	2.43	1.70E-03	ENSG00000115468
ENSBTAG00000027444	*SVIL*	2.37	4.98E-02	ENSG00000197321
ENSBTAG00000054434	(Non-annotated gene)	2.08	3.33E-02	No orthologue
ENSBTAG00000033429	*FAM229B*	1.96	4.92E-02	ENSG00000203778
ENSBTAG00000049950	(Non-annotated gene)	1.86	4.92E-02	No orthologue
ENSBTAG00000026758	(Non-annotated gene)	1.83	2.60E-02	ENSG00000211454
ENSBTAG00000017094	*SHMT1*	1.72	2.73E-02	ENSG00000176974
ENSBTAG00000038384	*KRT5*	1.48	1.21E-02	ENSG00000186081
ENSBTAG00000054234	(Non-annotated gene)	1.47	2.86E-02	ENSG00000178934
ENSBTAG00000003568	*CLDN10*	1.43	4.44E-03	ENSG00000134873
ENSBTAG00000004386	*SOCS1*	1.39	4.30E-03	ENSG00000185338
ENSBTAG00000012511	*BAD*	1.36	1.72E-03	ENSG00000002330
ENSBTAG00000003043	*GNG2*	1.33	2.09E-02	ENSG00000186469
ENSBTAG00000006086	*MMP28*	1.33	1.72E-03	ENSG00000271447
ENSBTAG00000022028	*DERL3*	1.17	2.52E-03	ENSG00000099958
ENSBTAG00000013922	*MOSPD1*	1.15	4.47E-02	ENSG00000101928
ENSBTAG00000020528	*PCOLCE*	1.12	1.01E-02	ENSG00000106333
ENSBTAG00000003222	*ASNS*	1.00	4.18E-02	ENSG00000070669
ENSBTAG00000010740	*CLTB*	0.88	4.21E-02	ENSG00000175416
ENSBTAG00000052249	(Non-annotated gene)	0.80	2.60E-02	ENSG00000204531
ENSBTAG00000021111	*POU5F1*	0.77	2.26E-03	ENSG00000204531
ENSBTAG00000017932	*CCDC84*	0.65	3.75E-02	ENSG00000186166

(A) Embryo ID 6HF

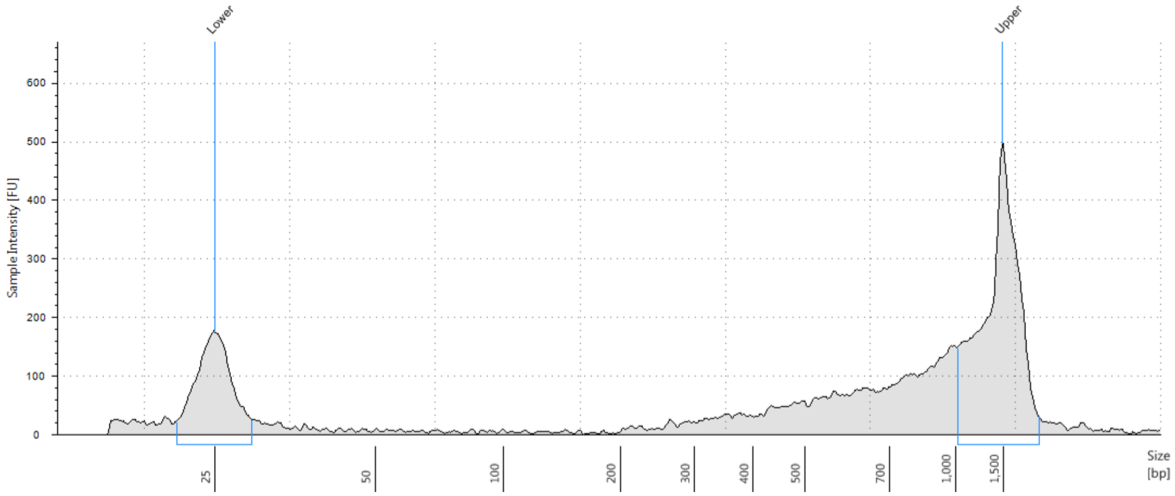

(B) Embryo ID 7HF

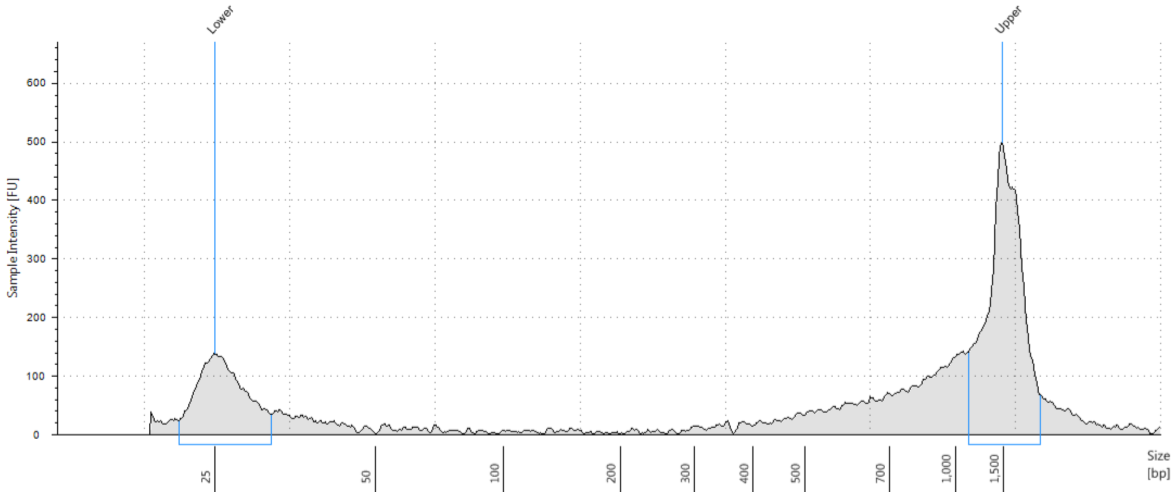

(C) Embryo ID 9HF

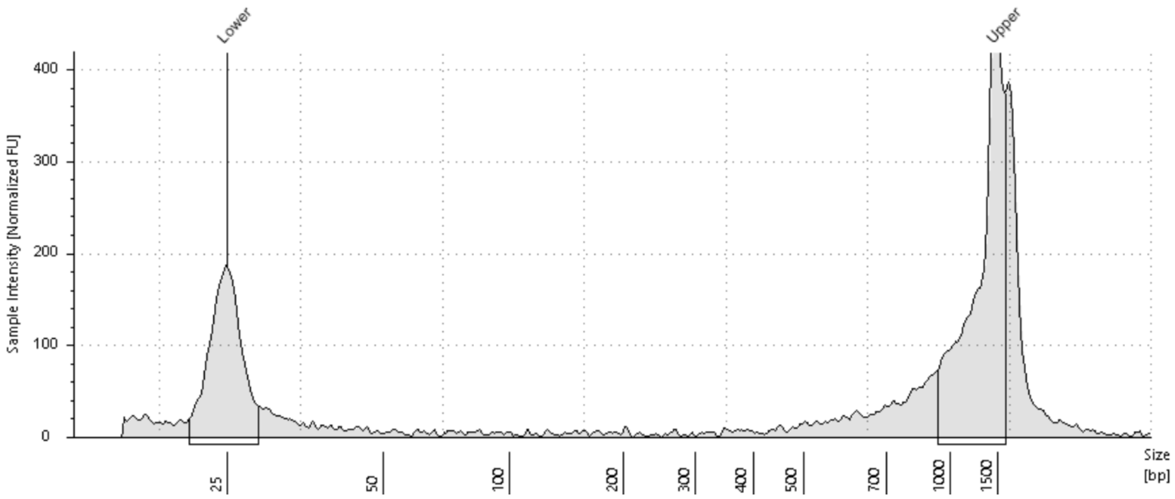

(D) Embryo ID 22HF

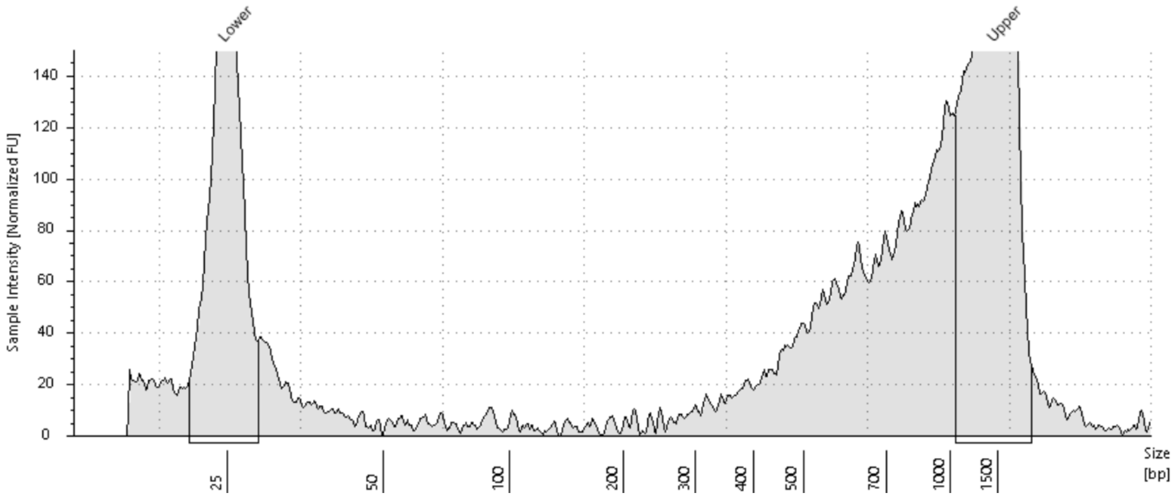

(E) Embryo ID 23HF

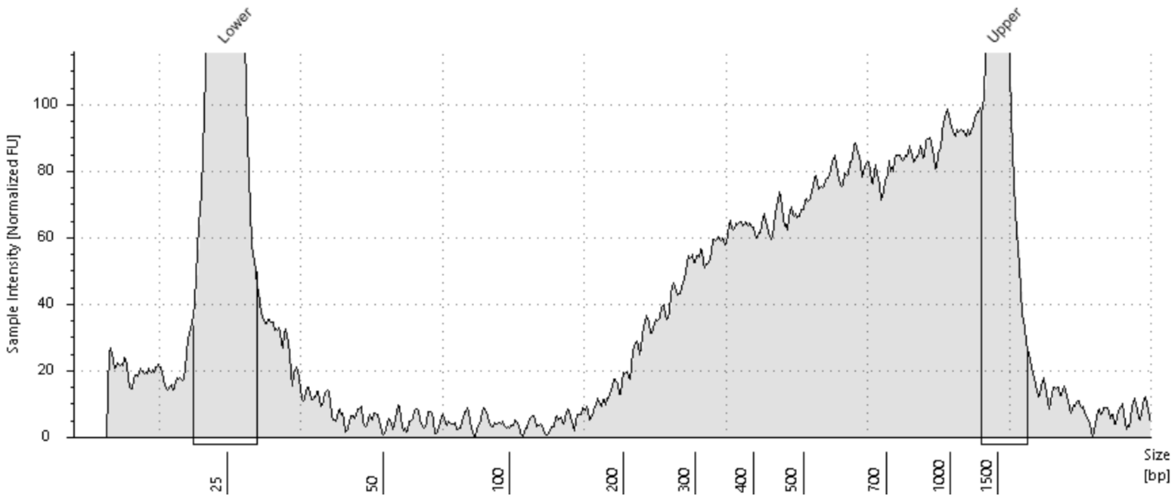

(F) Embryo ID 25HF

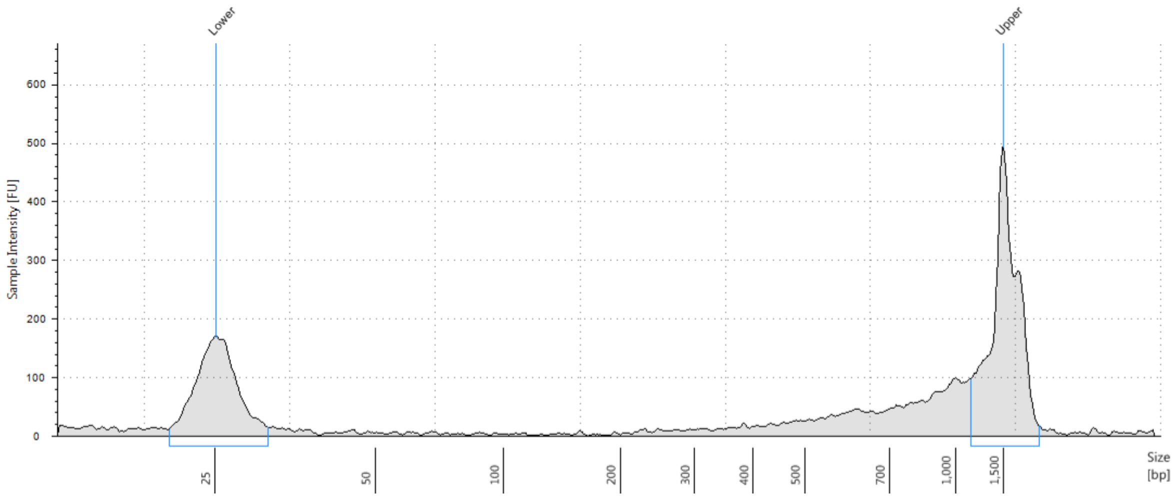

(G) Embryo ID 27HF

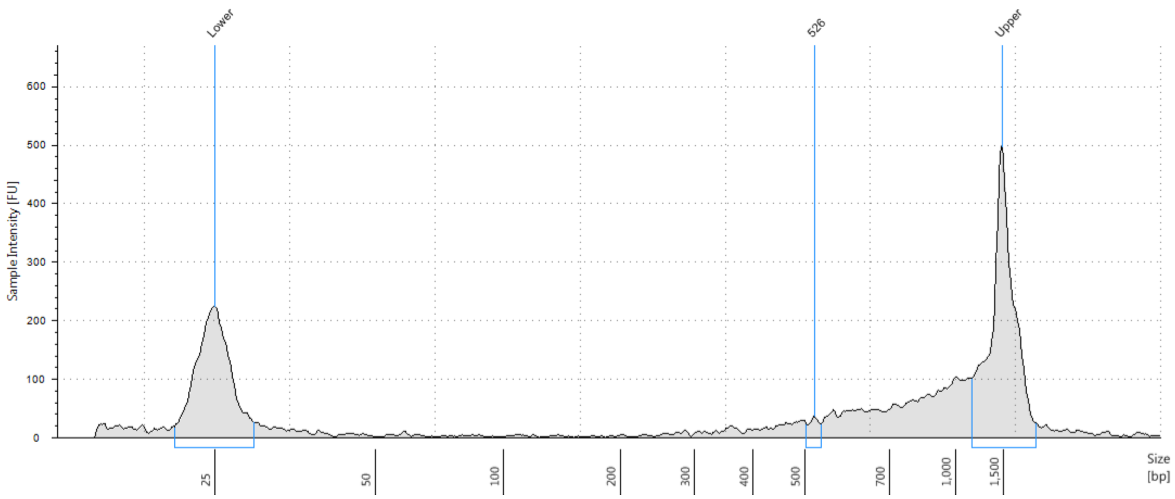

(H) Embryo ID 29HF

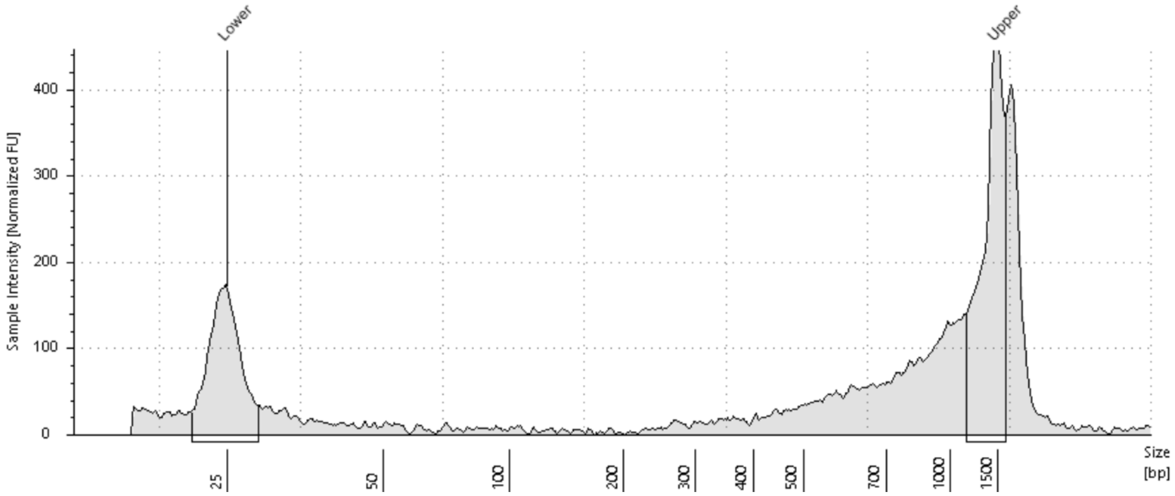

(I) Embryo ID 31HF

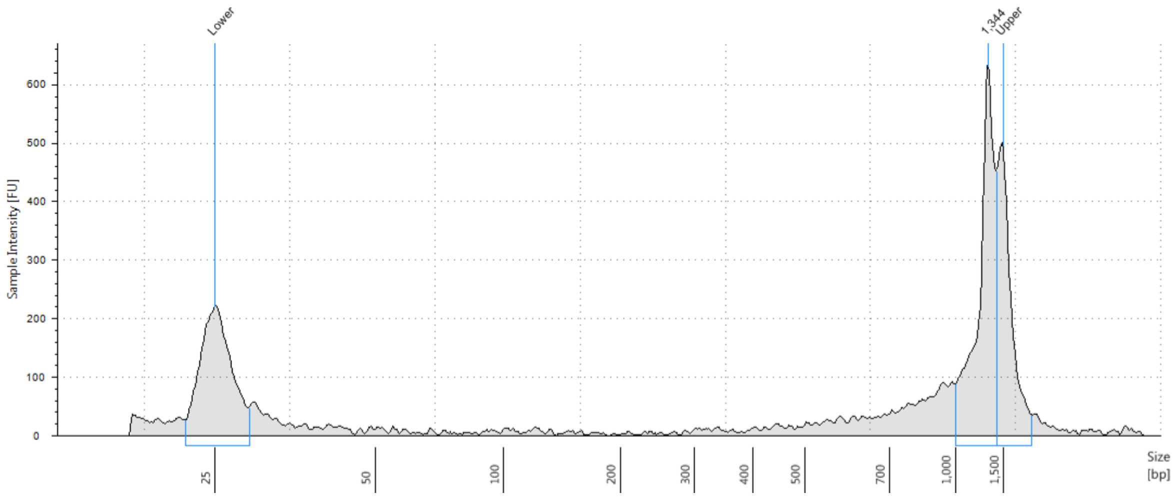

(J) Embryo ID 32HF

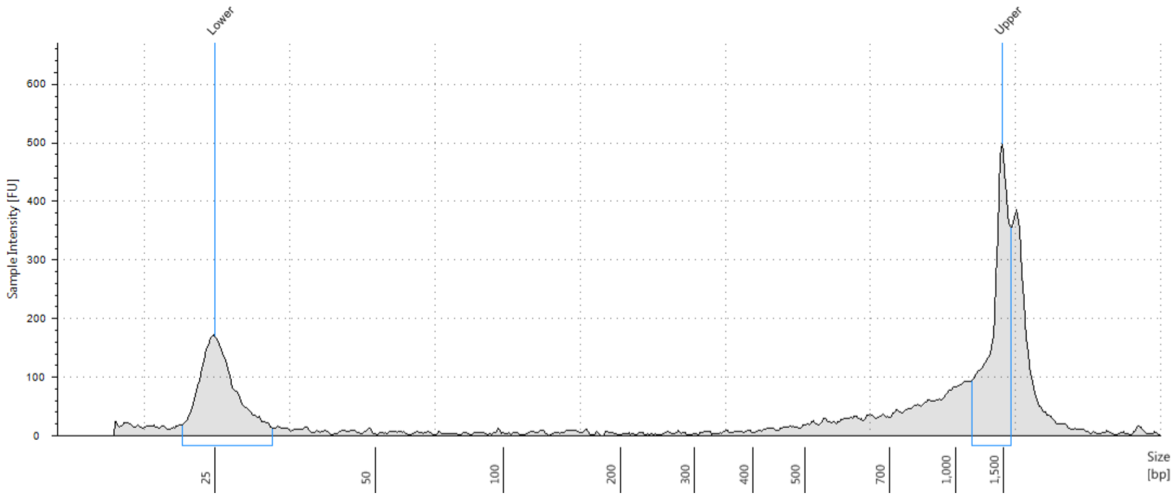

(K) Embryo ID 35HF

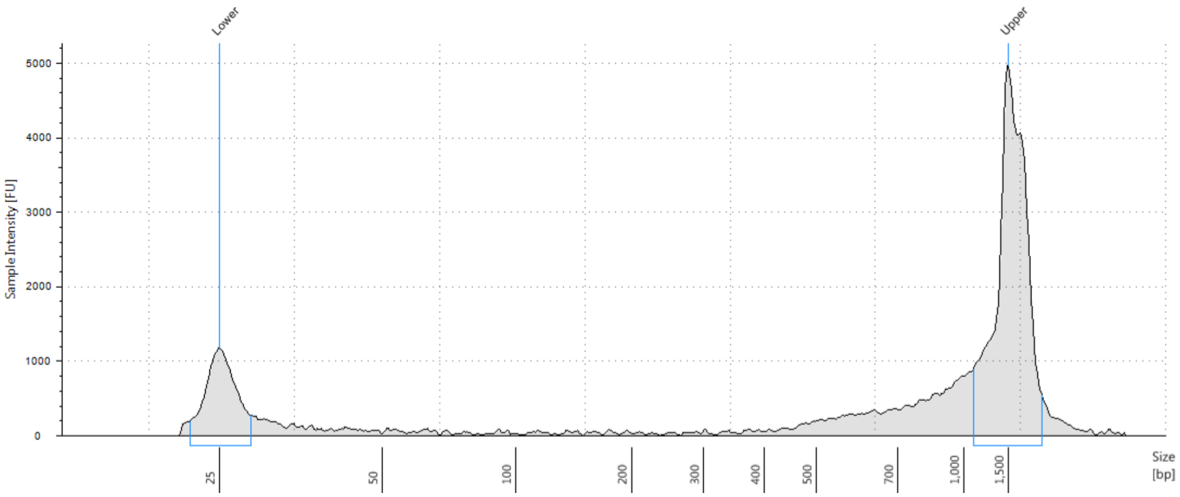

(L) Embryo ID 41HF

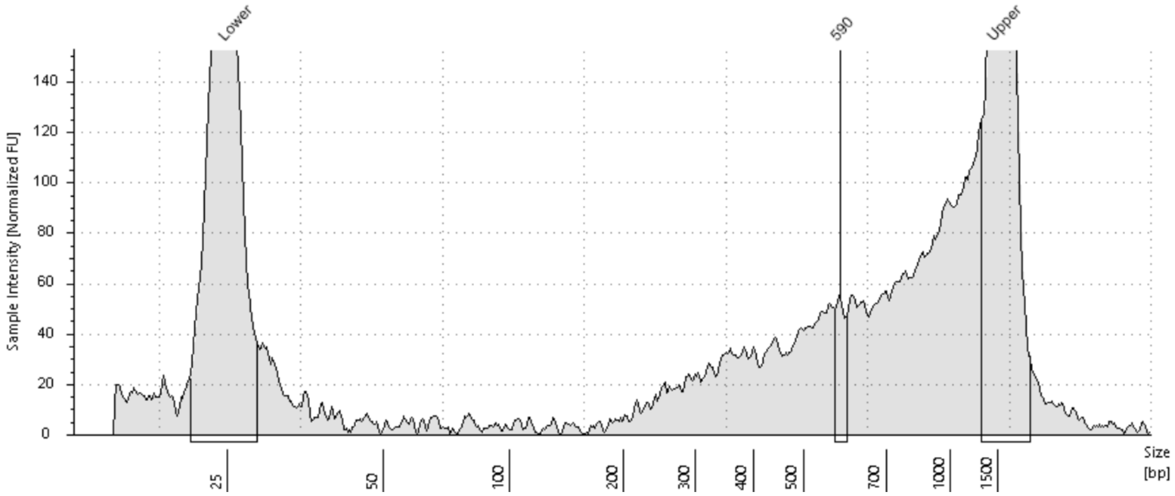

(M) Embryo ID 42HF

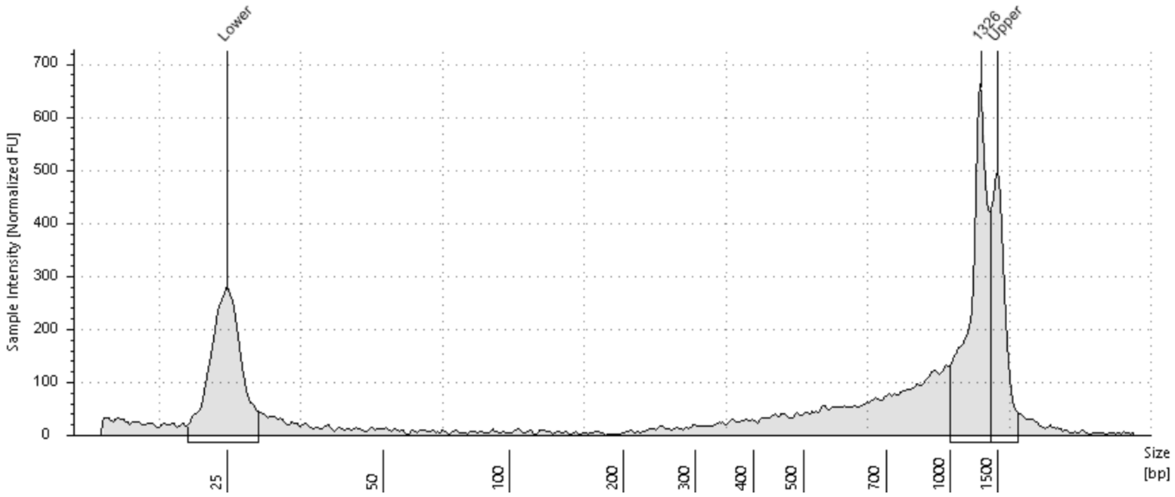

(N) Embryo ID 13LF

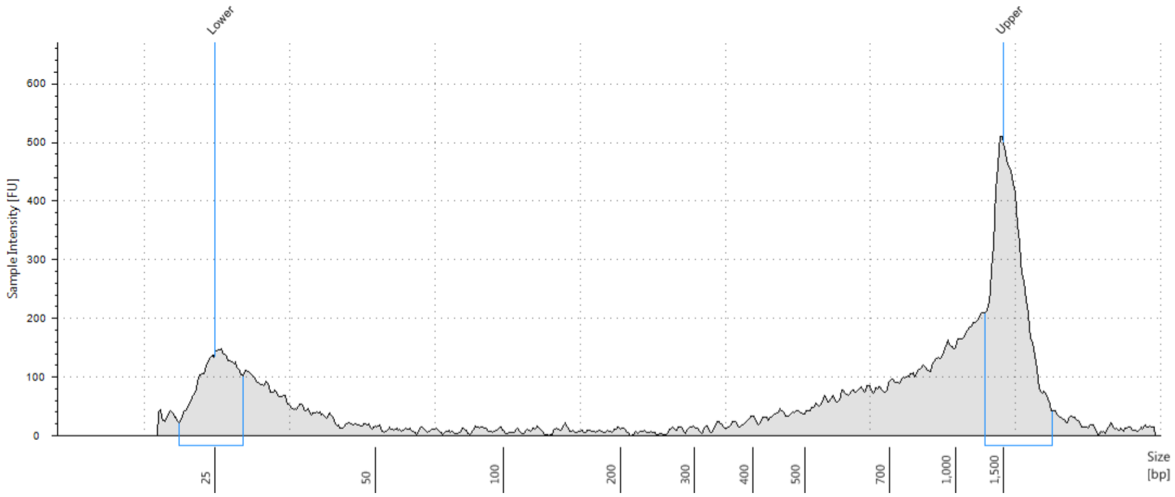

(O) Embryo ID 14LF

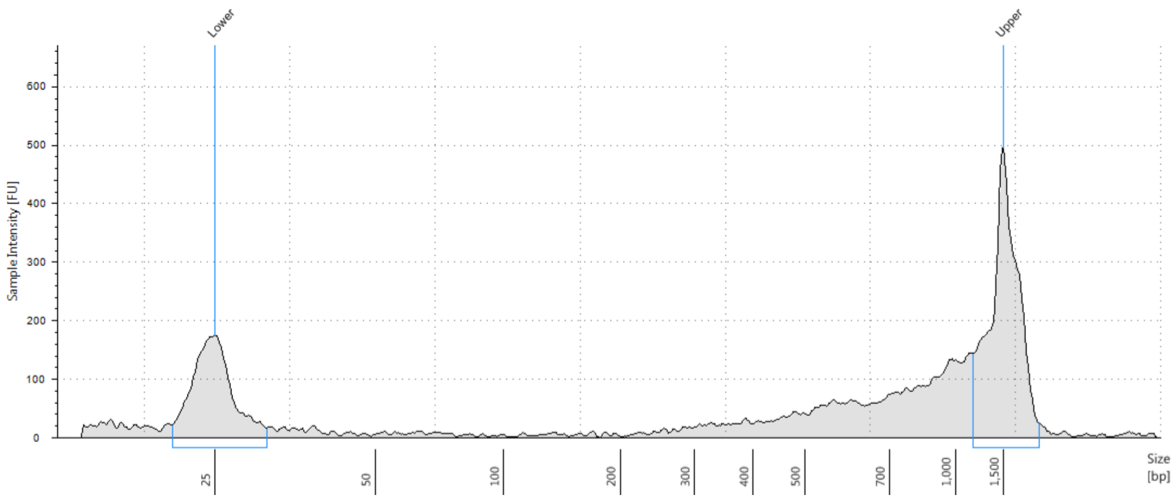

(P) Embryo ID 48LF*

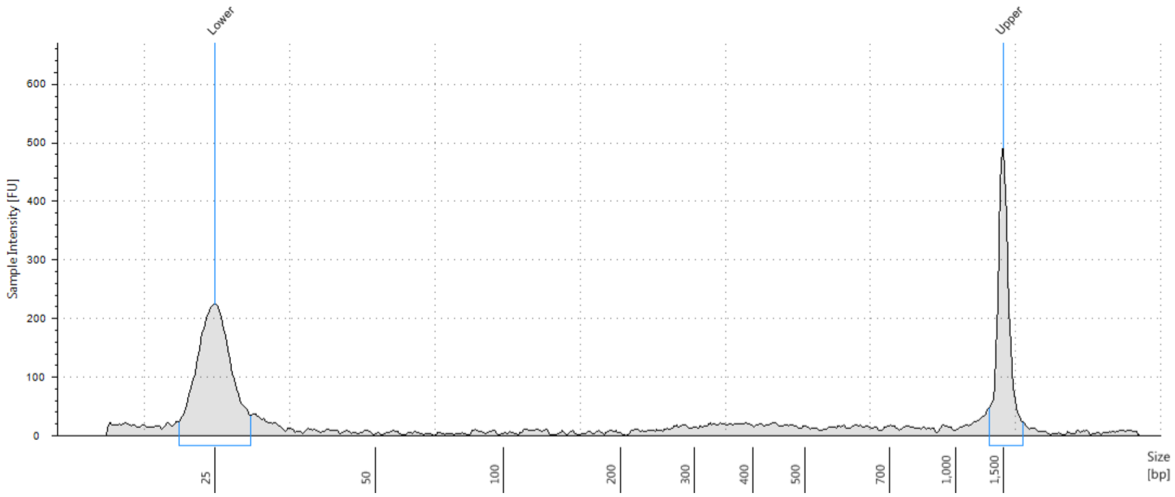

(Q) Embryo ID 49LF

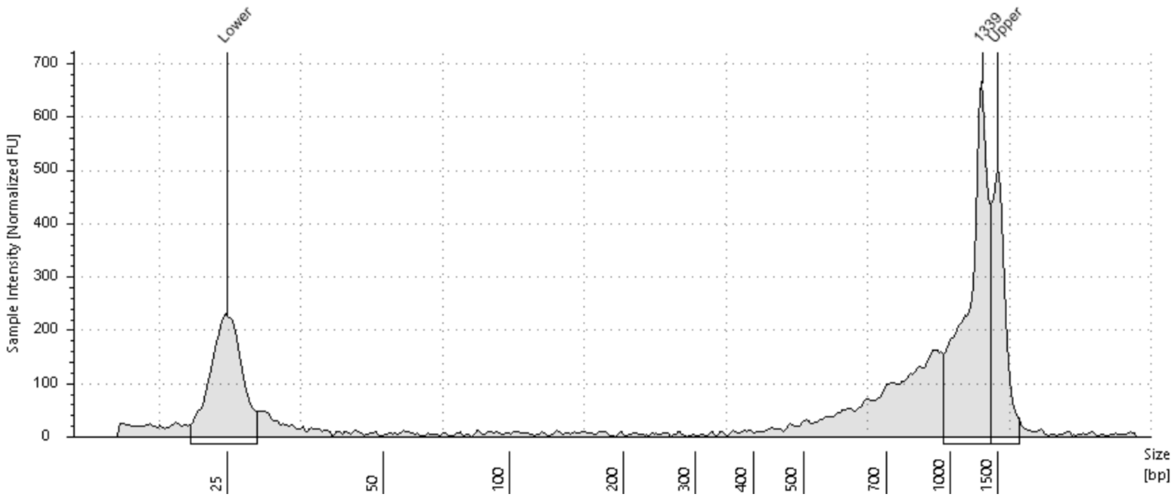

(R) Embryo ID 50LF

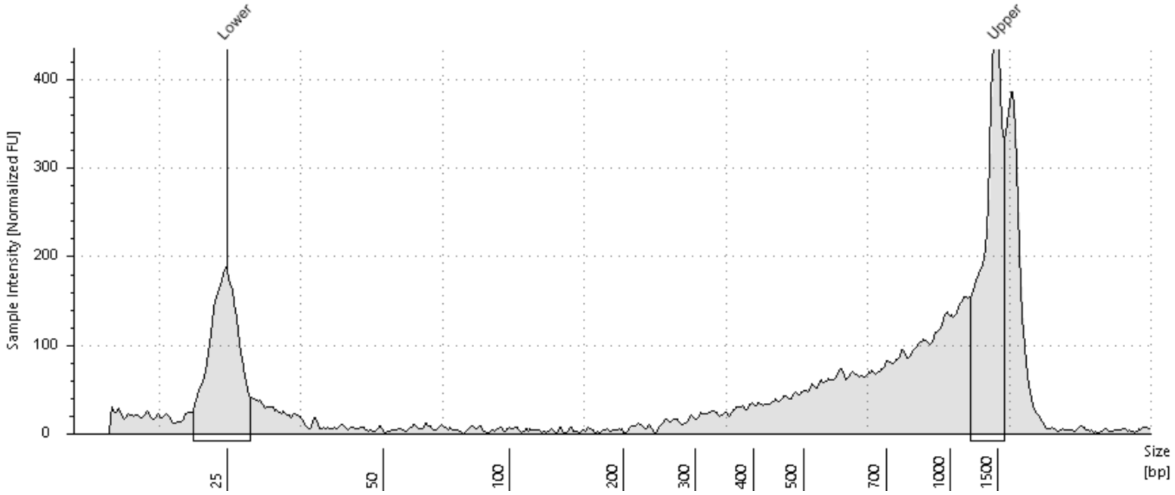

(S) Embryo ID 51LF

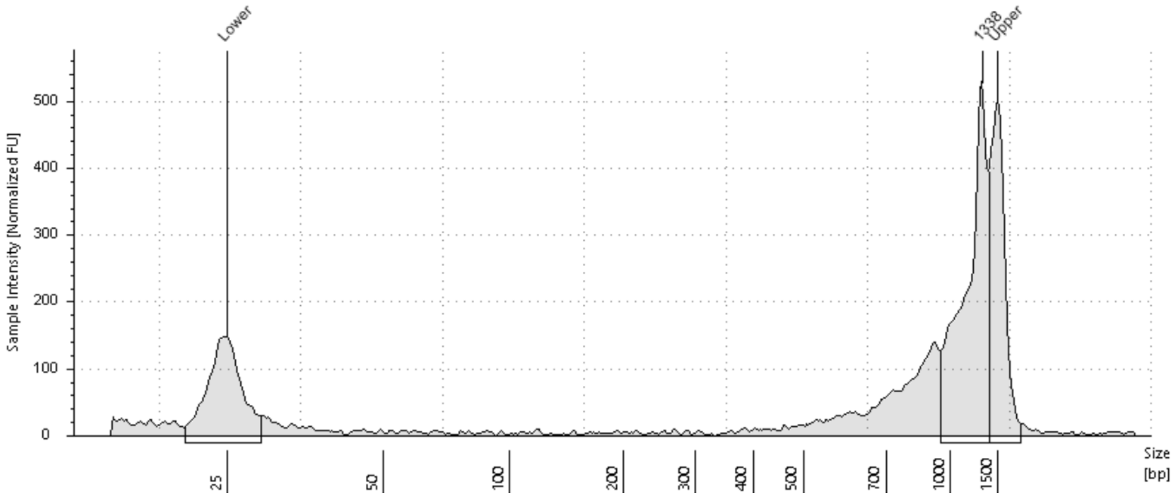

(T) Embryo ID 52LF

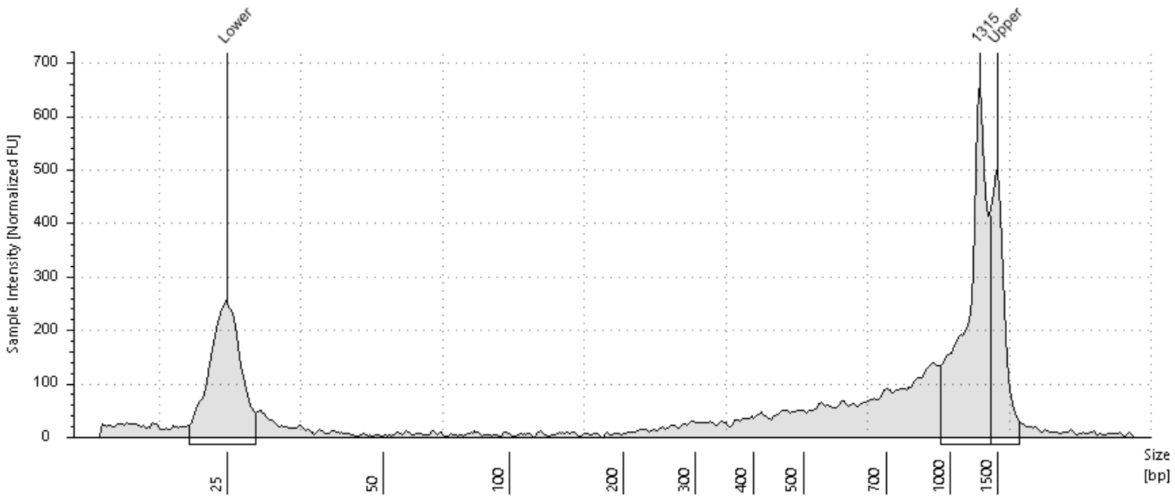

(U) Embryo ID 53LF

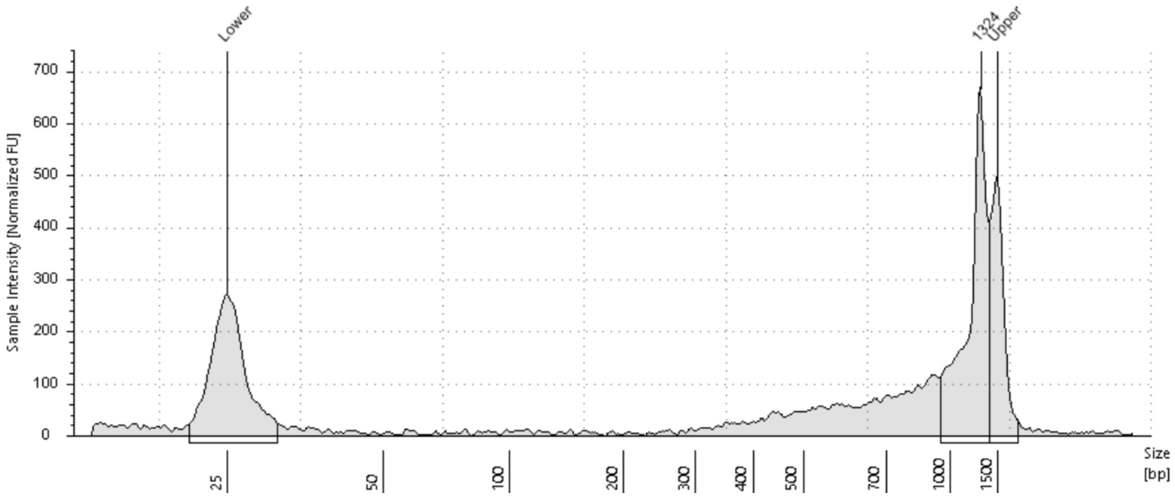

(V) Embryo ID 57LF

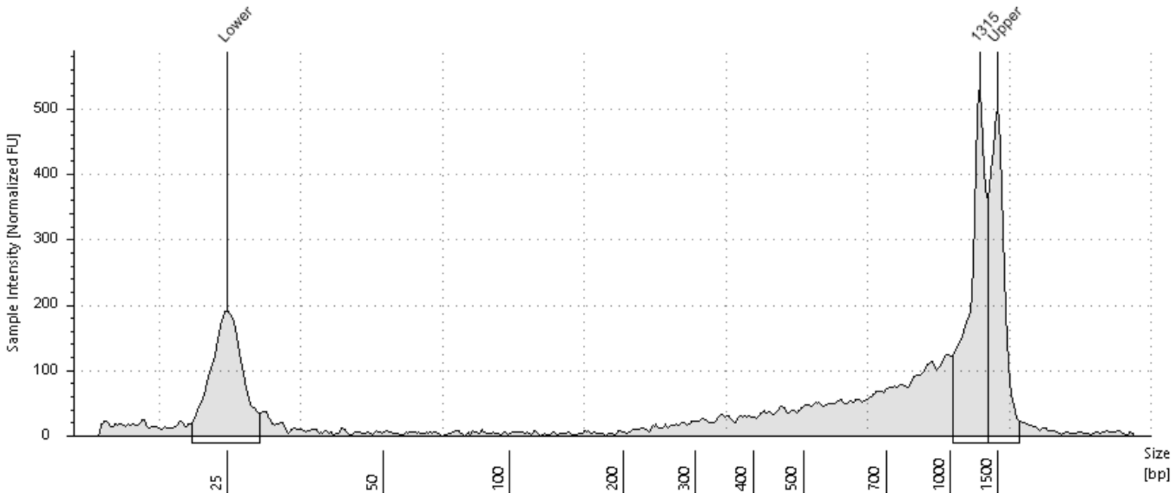

(W) Embryo ID 63LF

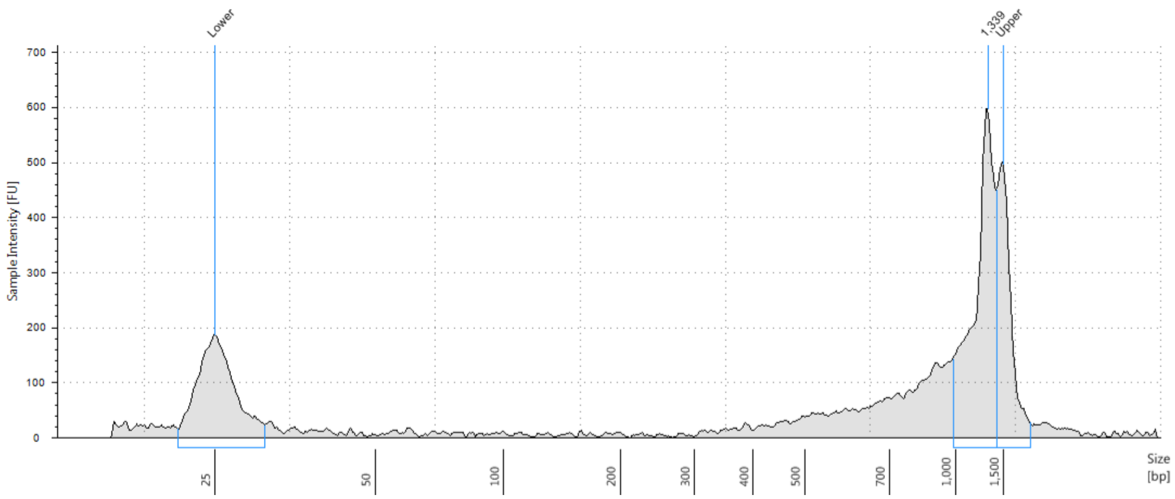

(X) Embryo ID 66LF*

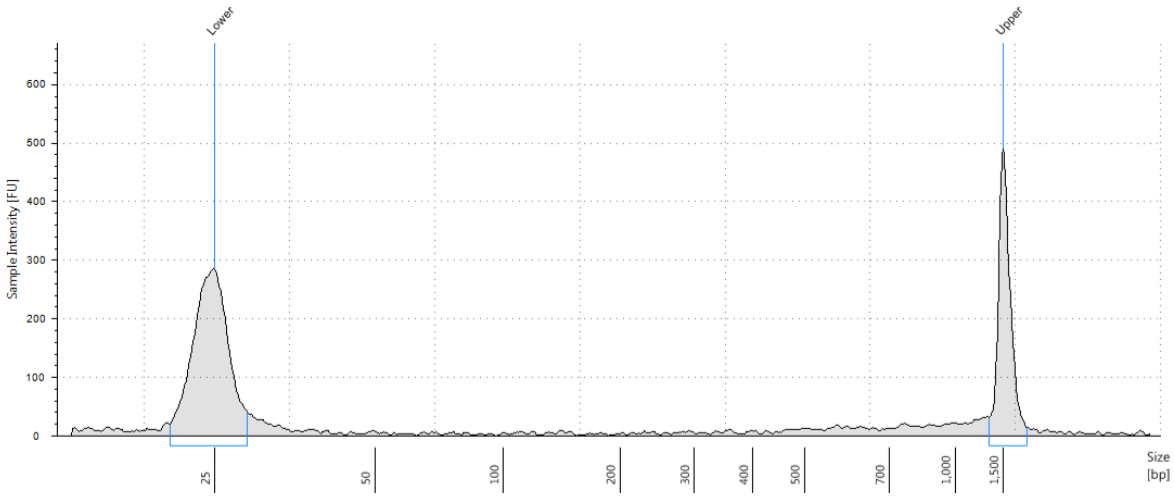

Supplementary figure 1: TapeStation profiles for all sequenced embryos. *These samples were not included in the DE-analysis.

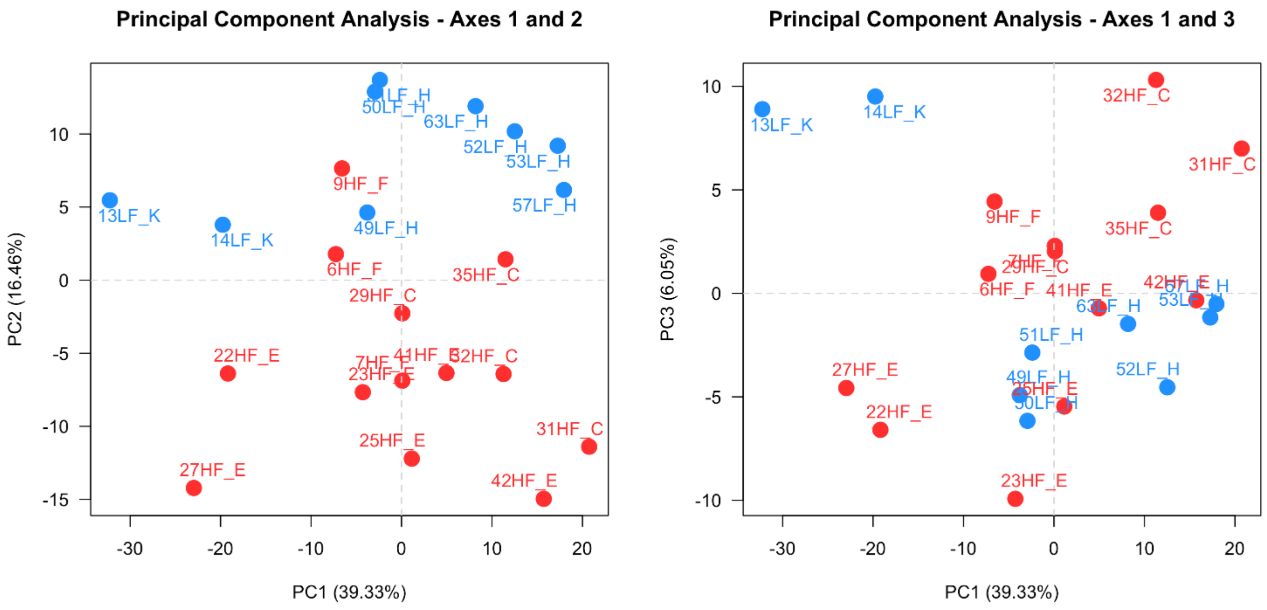

Supplementary Figure 2: Distribution of individual embryos, without embryo 48 and 66 which were considered outliers.

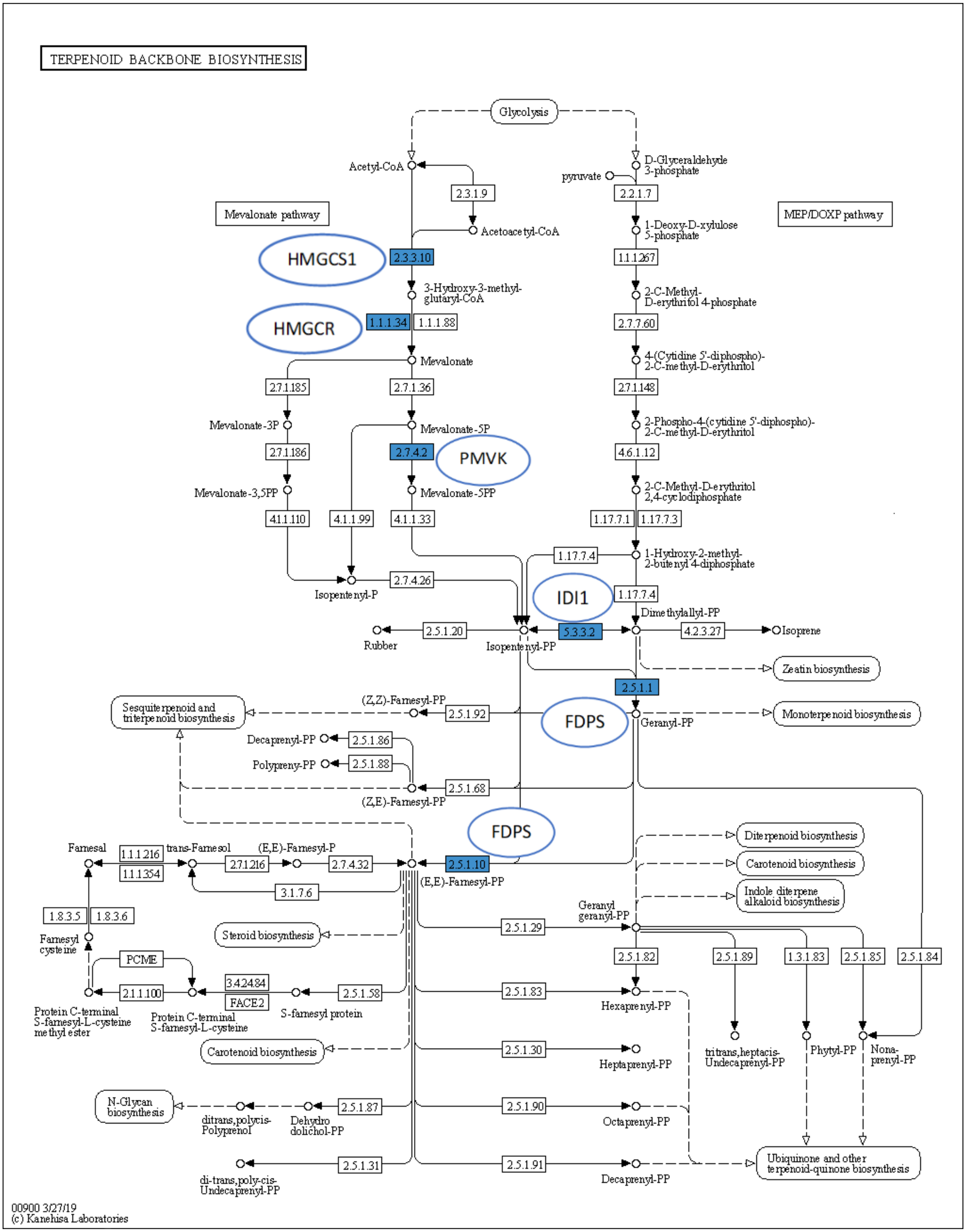
TERPENOID BACKBONE BIOSYNTHESIS
Glycolysis
Acetyl-CoA
D-Glyceraldehyde 3-phosphate
pyruvate
2.3.1.9
2.2.1.7
Mevalonate pathway
MEP/DOXP pathway
Acetoacetyl-CoA
1-Deoxy-D-xylulose 5-phosphate
HMGCS1
2.3.3.10
1.1.1.267
3-Hydroxy-3-methyl-glutaryl-CoA
2-C-Methyl-D-erythritol 4-phosphate
HMGCR
1.1.1.34
1.1.1.88
2.7.7.60
Mevalonate
4-(Cytidine 5'-diphospho)-2-C-methyl-D-erythritol
2.7.1.185
2.7.1.36
2.7.1.148
Mevalonate-3P
Mevalonate-5P
2-Phospho-4-(cytidine 5'-diphospho)-2-C-methyl-D-erythritol
2.7.1.186
2.7.4.2
PMVK
4.6.1.12
Mevalonate-3,5PP
Mevalonate-5PP
2-C-Methyl-D-erythritol 2,4-cyclodiphosphate
4.1.1.110
4.1.1.99
4.1.1.33
1.17.7.1
1.17.7.3
1.17.7.4
1-Hydroxy-2-methyl-2-butenyl 4-diphosphate
2.7.4.26
IDI1
1.17.7.4
Isopentenyl-P
Dimethylallyl-PP
2.5.1.20
5.3.3.2
4.2.3.27
Isoprene
Rubber
Isopentenyl-PP
Zeatin biosynthesis
2.5.1.1
(Z,Z)-Farnesyl-PP
Sesquiterpenoid and triterpenoid biosynthesis
2.5.1.92
FDPS
Monoterpenoid biosynthesis
Geranyl-PP
Decaprenyl-PP
2.5.1.86
Polypreny-PP
2.5.1.88
2.5.1.68
(Z,E)-Farnesyl-PP
FDPS
Diterpenoid biosynthesis
Farnesal
1.1.1.216
trans-Farnesol
(E,E)-Farnesyl-P
1.1.1.354
2.7.1.216
2.7.4.32
2.5.1.10
(E,E)-Farnesyl-PP
Carotenoid biosynthesis
3.1.7.6
Indole diterpene alkaloid biosynthesis
1.8.3.5
1.8.3.6
Geranyl geranyl-PP
Farnesyl cysteine
Steroid biosynthesis
2.5.1.29
PCME
2.5.1.82
2.5.1.89
1.3.1.83
2.5.1.85
2.5.1.84
3.4.24.84
2.1.1.100
FACE2
2.5.1.58
2.5.1.83
Hexaprenyl-PP
Protein C-terminal S-farnesyl-L-cysteine methyl ester
Protein C-terminal S-farnesyl-L-cysteine
S-farnesyl protein
tritrans,heptacis-Undecaprenyl-PP
Phytyl-PP
Nona-prenyl-PP
Carotenoid biosynthesis
2.5.1.30
Heptaprenyl-PP
N-Glycan biosynthesis
2.5.1.87
2.5.1.90
ditrans,polycis-Polyprenol
Dehydro dolichol-PP
Octaprenyl-PP
Ubiquinone and other terpenoid-quinone biosynthesis
2.5.1.31
2.5.1.91
di-trans,poly-cis-Undecaprenyl-PP
Decaprenyl-PP
00900 3/27/19
(c) Kanehisa Laboratories

Supplementary Figure 3: Terpenoid backbone biosynthesis. Gene products that were highly expressed in the LF embryos are marked in blue. Gene symbols are marked in circles. Modified from KEGG chart bta00900, 3/27/19 Kaneshia Laboratories, URL: kegg.jp

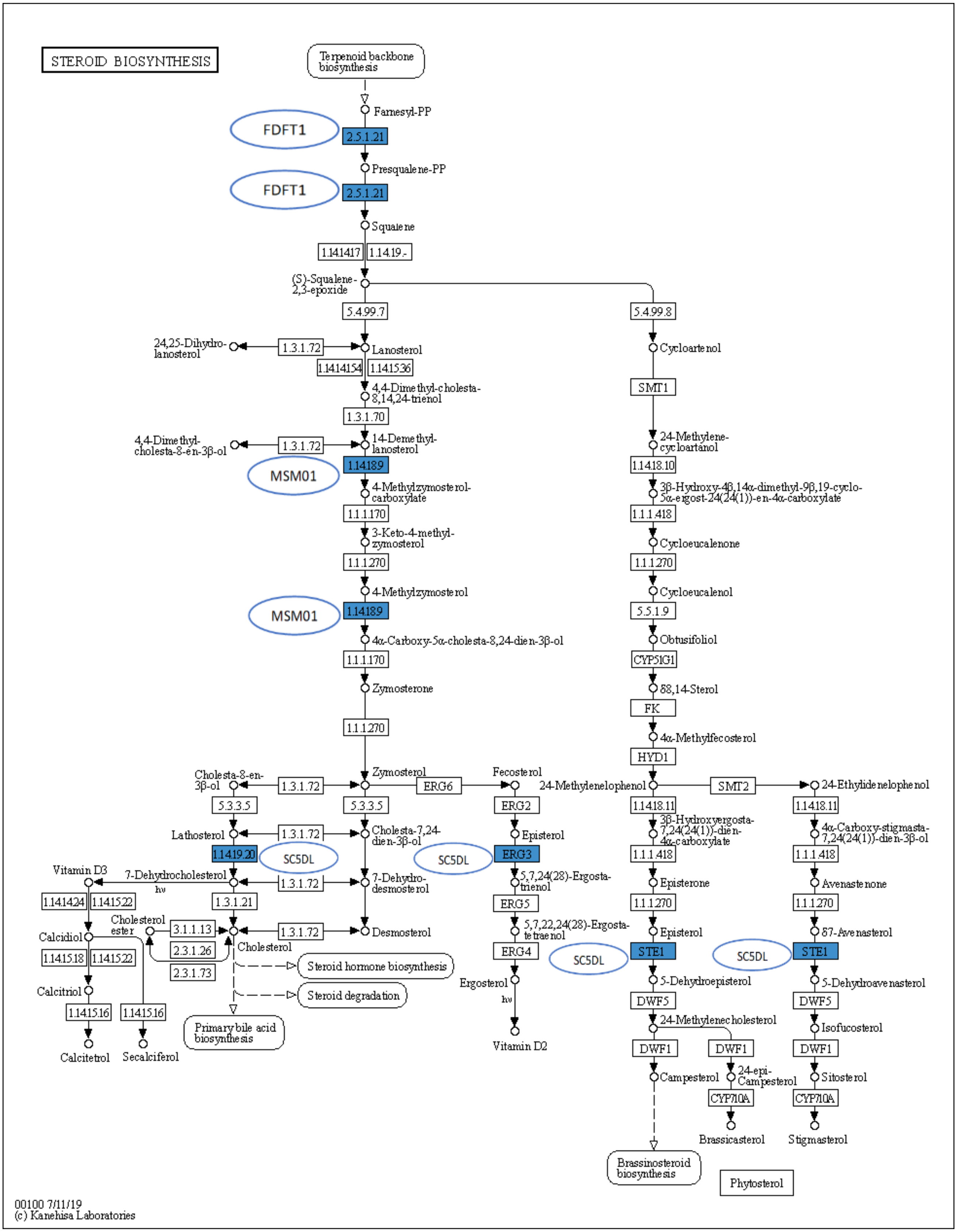
STEROID BIOSYNTHESIS
Terpenoid backbone biosynthesis
Farnesyl-PP
FDFT1
2.5.1.21
Presqualene-PP
Squalene
1.14.14.17
1.14.19.-
(S)-Squalene-2,3-epoxide
5.4.99.7
5.4.99.8
Lanosterol
Cycloartenol
24,25-Dihydro-lanosterol
1.3.1.72
1.14.14.154
1.14.15.36
SMT1
4,4-Dimethyl-cholesta-8,14,24-trienol
1.3.1.70
4,4-Dimethyl-cholesta-8-en-3β-ol
14-Demethyl-lanosterol
24-Methylene-cycloartanol
MSMO1
1.14.18.9
1.14.18.10
4-Methylzymosterol-carboxylate
1.1.1.170
3-Keto-4-methyl-zymosterol
1.1.1.270
4-Methylzymosterol
Cycloeucalenone
Cycloeucalenol
5.5.1.9
Obtusifoliol
CYP51G1
4α-Carboxy-5α-cholesta-8,24-dien-3β-ol
Zymosterone
δ8,14-Sterol
FK
4α-Methylfecosterol
HYD1
Zymosterol
Fecosterol
ERG6
24-Methylenelophenol
SMT2
24-Ethylidenelophenol
Cholesta-8-en-3β-ol
5.3.3.5
ERG2
1.14.18.11
Lathosterol
Cholesta-7,24-dien-3β-ol
Episterol
4α-Carboxy-stigmasta-7,24(24(1))-dien-3β-ol
1.14.19.20
SC5DL
ERG3
1.1.1.418
Vitamin D3
7-Dehydrocholesterol
hν
7-Dehydro-desmosterol
5,7,24(28)-Ergosta-trienol
Episterone
Avenastenone
1.14.14.24
1.14.15.22
1.3.1.21
ERG5
Cholesterol ester
3.1.1.13
Calcidiol
Desmosterol
5,7,22,24(28)-Ergosta-tetraenol
δ7-Avenasterol
2.3.1.26
Cholesterol
ERG4
STE1
1.14.15.18
2.3.1.73
Steroid hormone biosynthesis
Calcitriol
Steroid degradation
Ergosterol
5-Dehydroepisterol
5-Dehydroavenasterol
DWF5
1.14.15.16
Primary bile acid biosynthesis
24-Methylenecholesterol
Isofucosterol
DWF1
Vitamin D2
Calcitetrol
Secalciferol
Campesterol
24-epi-Campesterol
Sitosterol
CYP710A
Brassicasterol
Stigmasterol
Brassinosteroid biosynthesis
Phytosterol
00100 7/11/19
(c) Kanehisa Laboratories

Supplementary Figure 4: Steroid biosynthesis. Gene products that were highly expressed in the LF embryos are marked in blue. Gene symbols are marked in circles. Modified from KEGG chart bta00100, 7/11/19 Kaneshia Laboratories, URL: kegg.jp

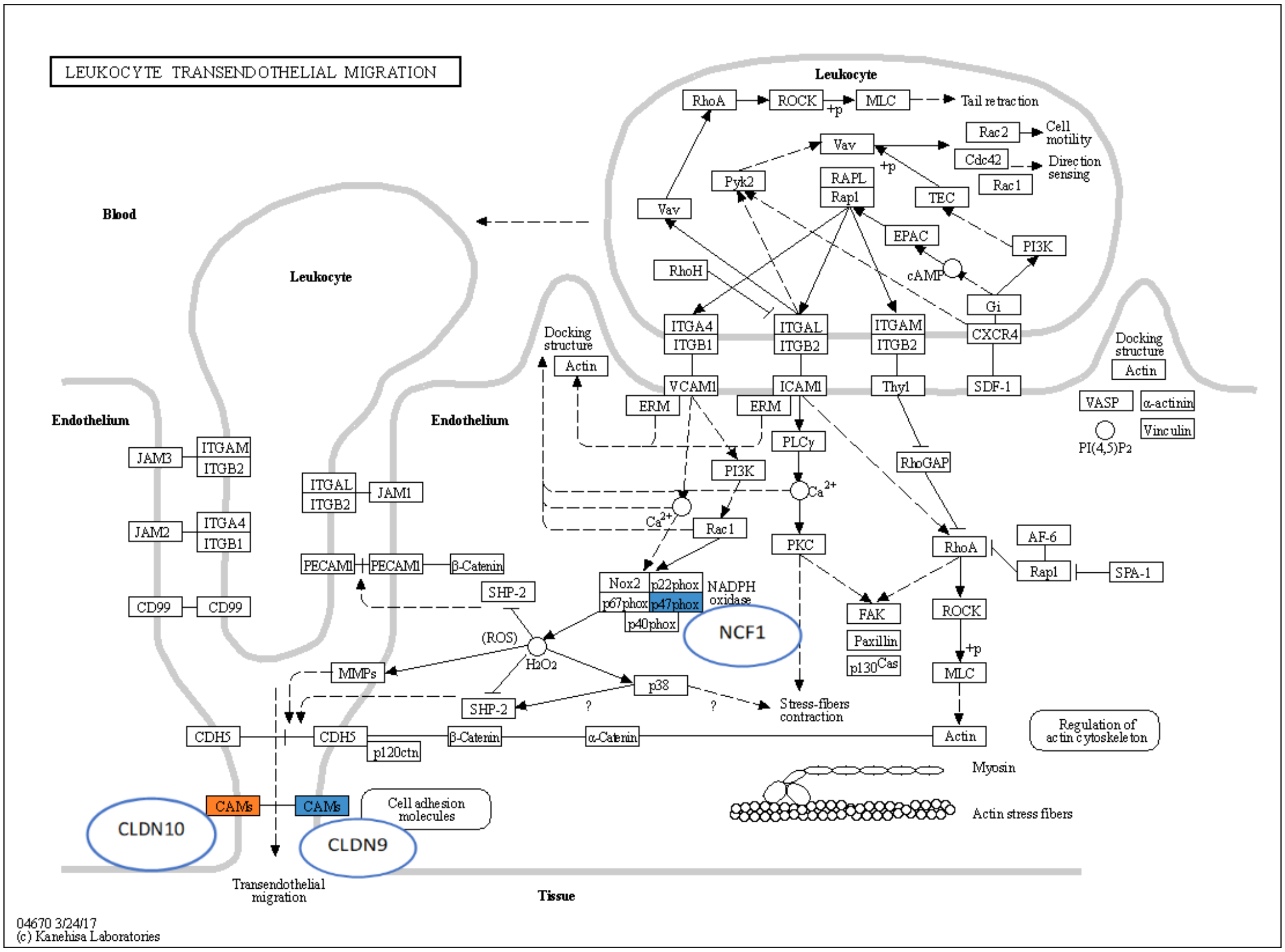

Supplementary Figure 5: Leukocyte transendothelial migration. Gene products that were highly expressed in the LF embryos are marked in blue, while gene products that were highly expressed in the HF embryos are marked in orange. Gene symbols are marked in circles. Modified from KEGG chart bta04670, 3/24/17 Kaneshia Laboratories, URL: kegg.jp

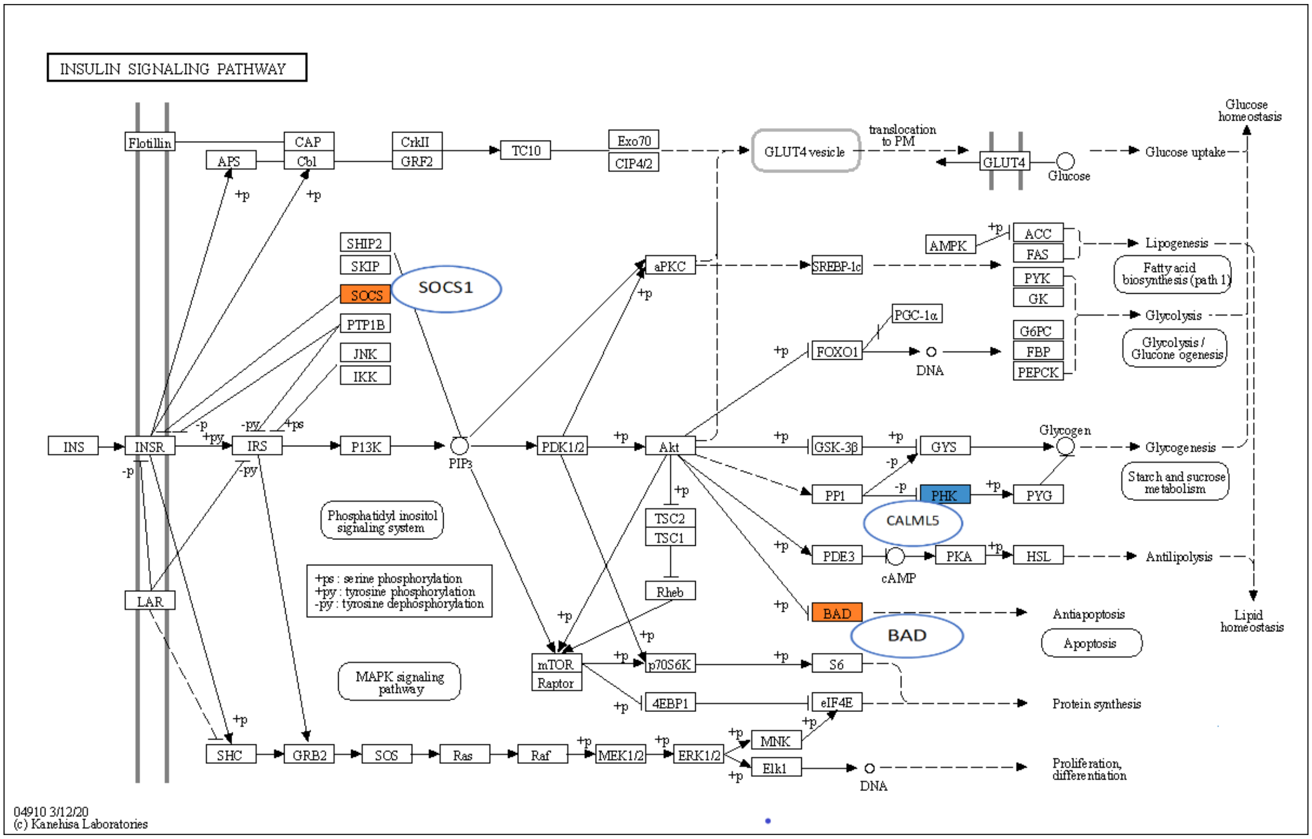

Supplementary Figure 6: Insulin signaling pathway. Gene products that were highly expressed in the LF embryos are marked in blue, while gene products that were highly expressed in the HF embryos are marked in orange. Gene symbols are marked in circles. Modified from KEGG chart bta04910, 3/12/20 Kaneshia Laboratories, URL: kegg.jp
