# Supplementary material for: Gene Expression in Embryos From Norwegian Red Bulls With High or Low Non Return Rate: An RNA-Seq Study of in vivo-Produced Single Embryos
Source: Front Genet. 2022 Jan 14;12:780113. doi: 10.3389/fgene.2021.780113 (PMC8795813; doi:10.3389/fgene.2021.780113)

(A) Embryo ID 6HF

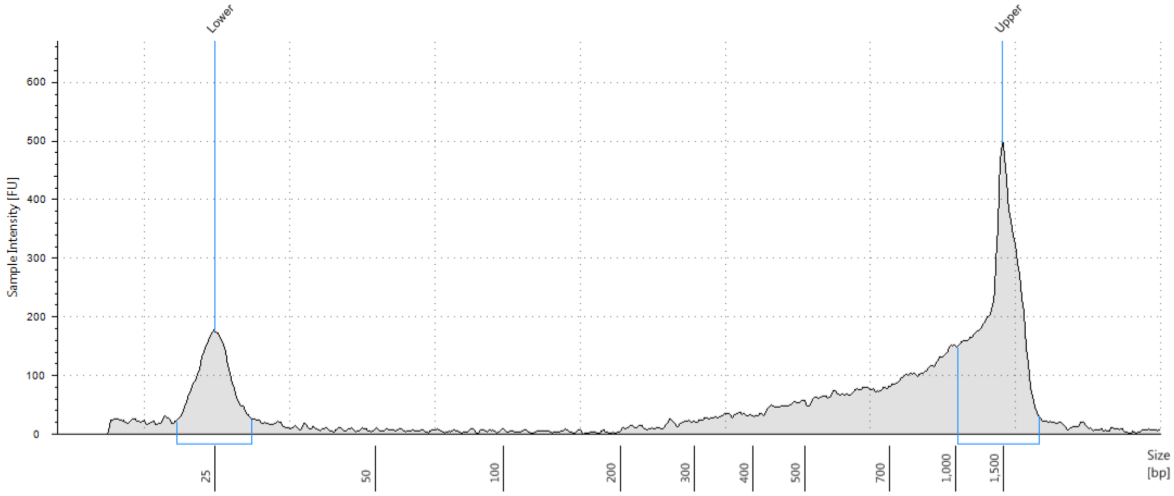

(B) Embryo ID 7HF

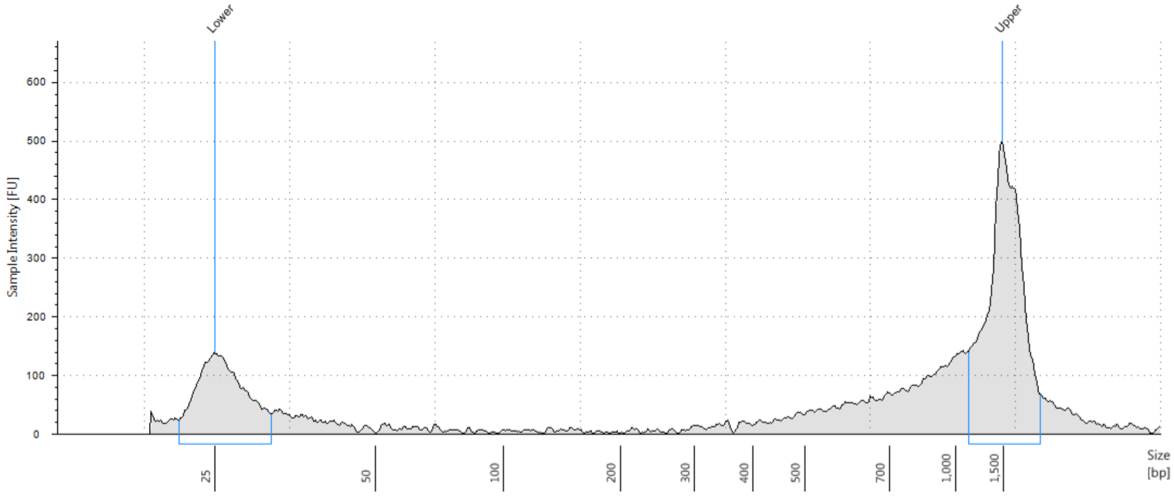

(C) Embryo ID 9HF

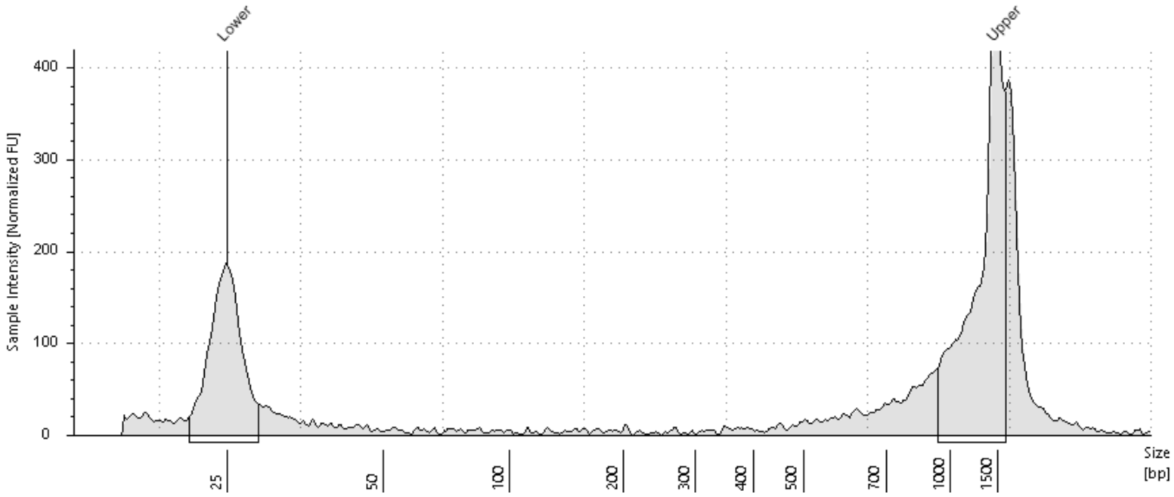

(D) Embryo ID 22HF

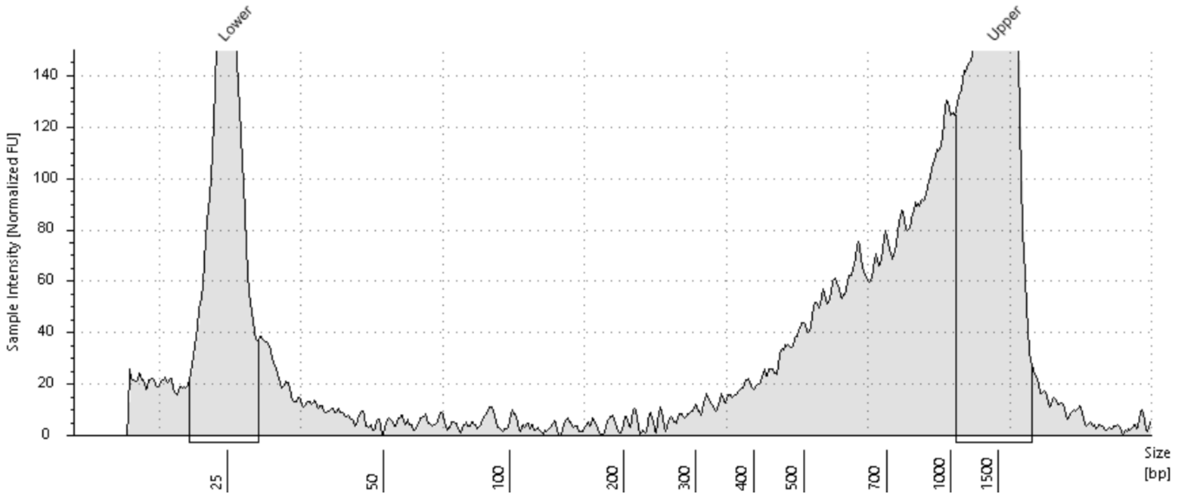

(E) Embryo ID 23HF

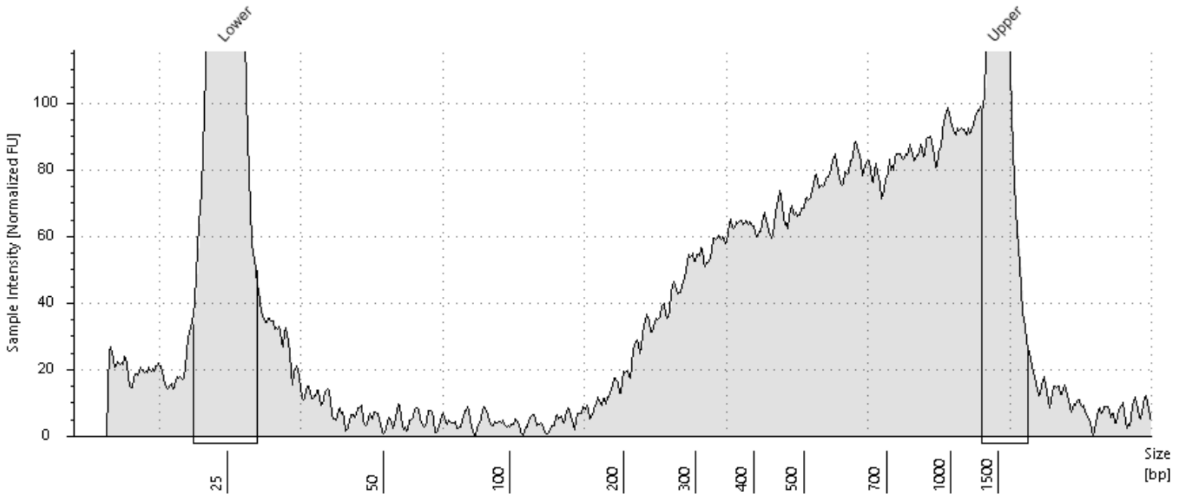

(F) Embryo ID 25HF

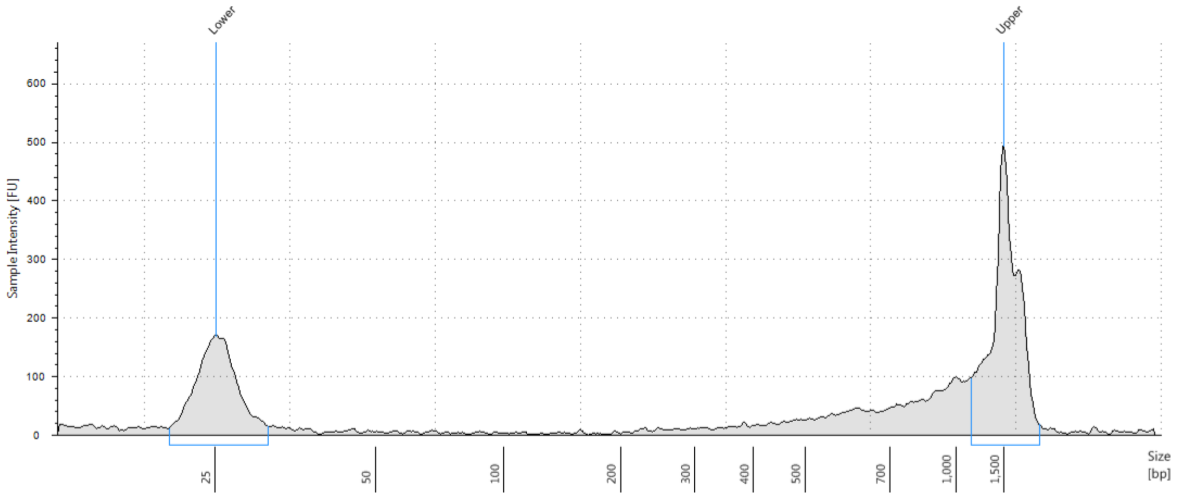

(G) Embryo ID 27HF

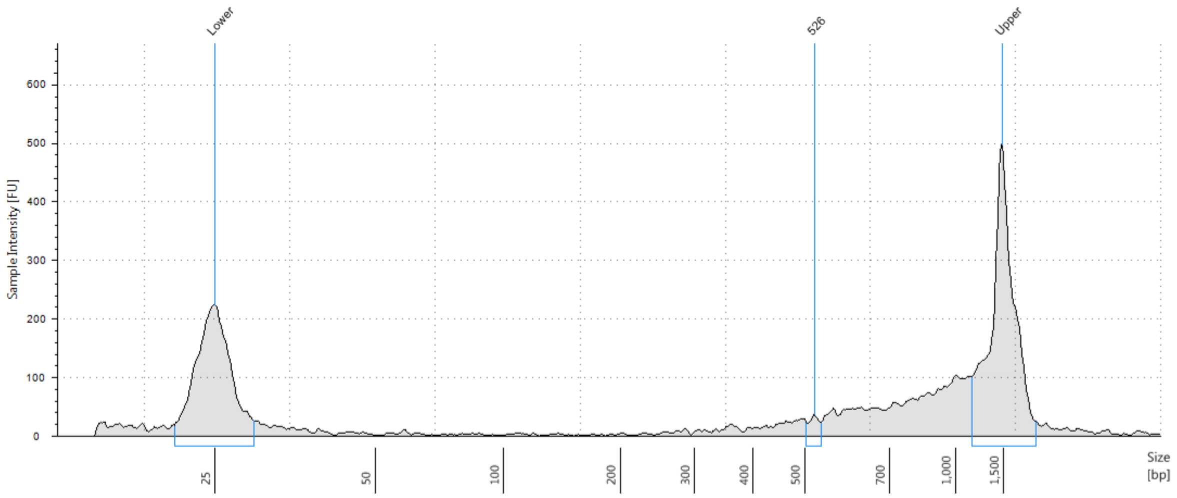

(H) Embryo ID 29HF

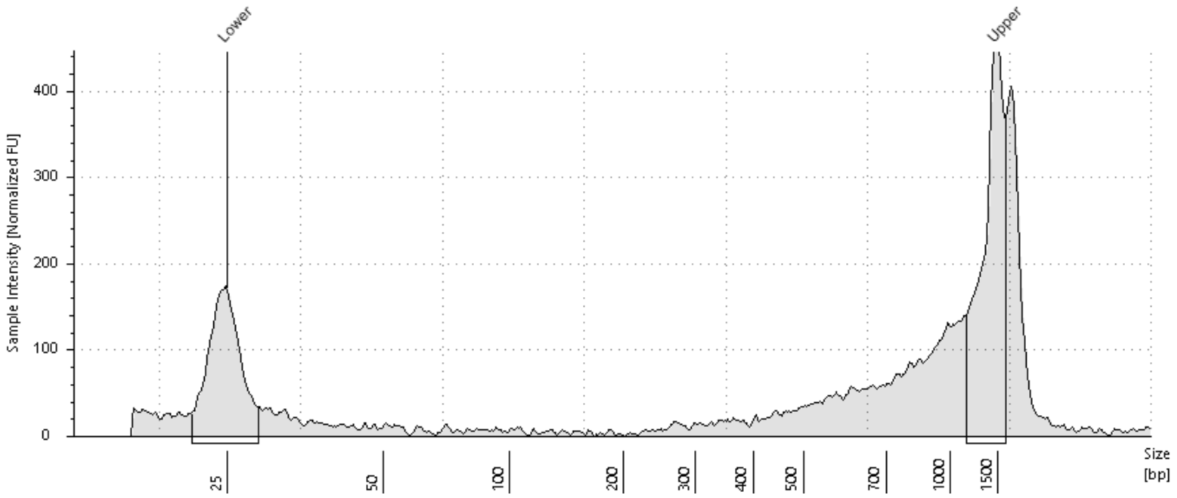

(I) Embryo ID 31HF

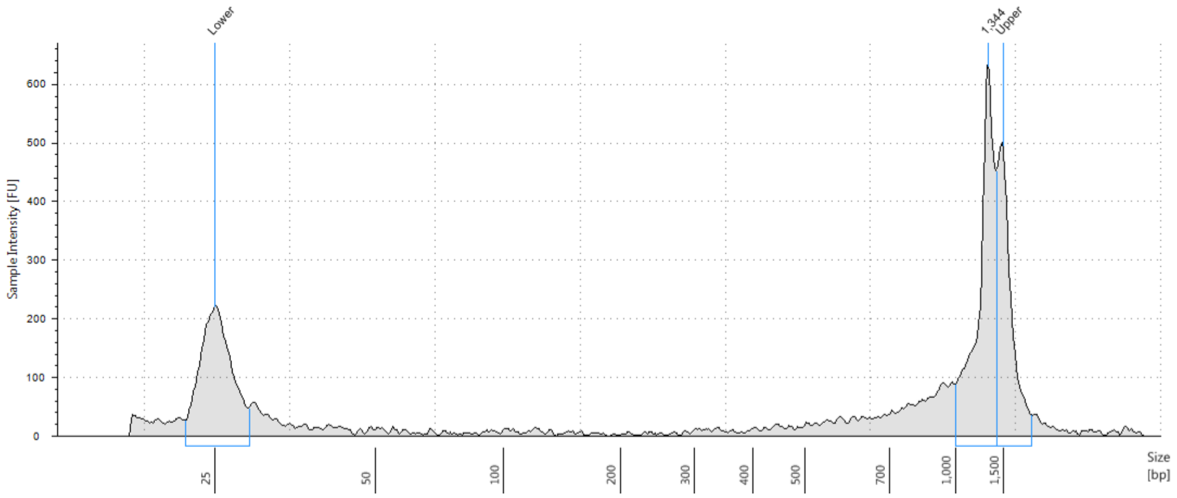

(J) Embryo ID 32HF

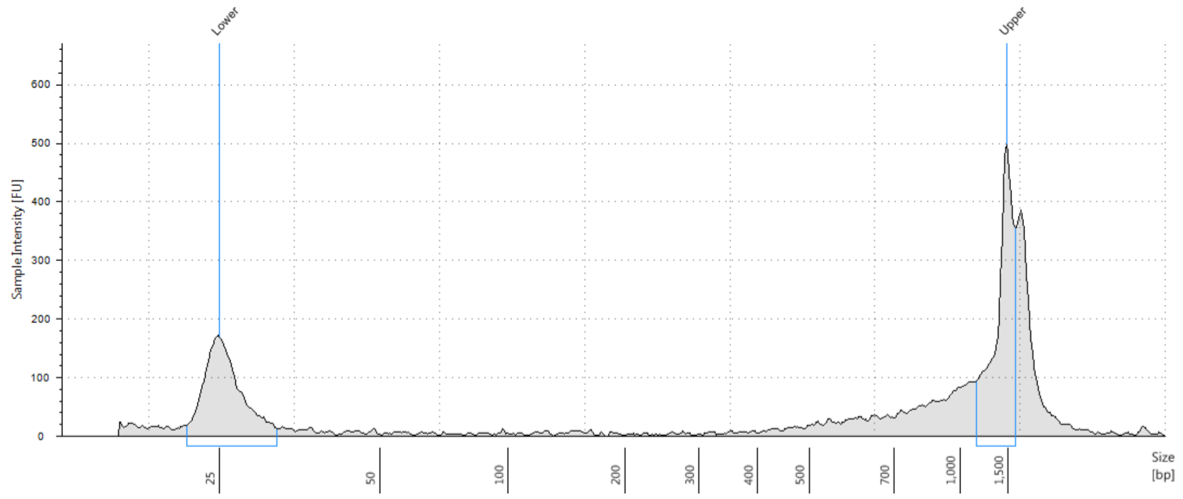

(K) Embryo ID 35HF

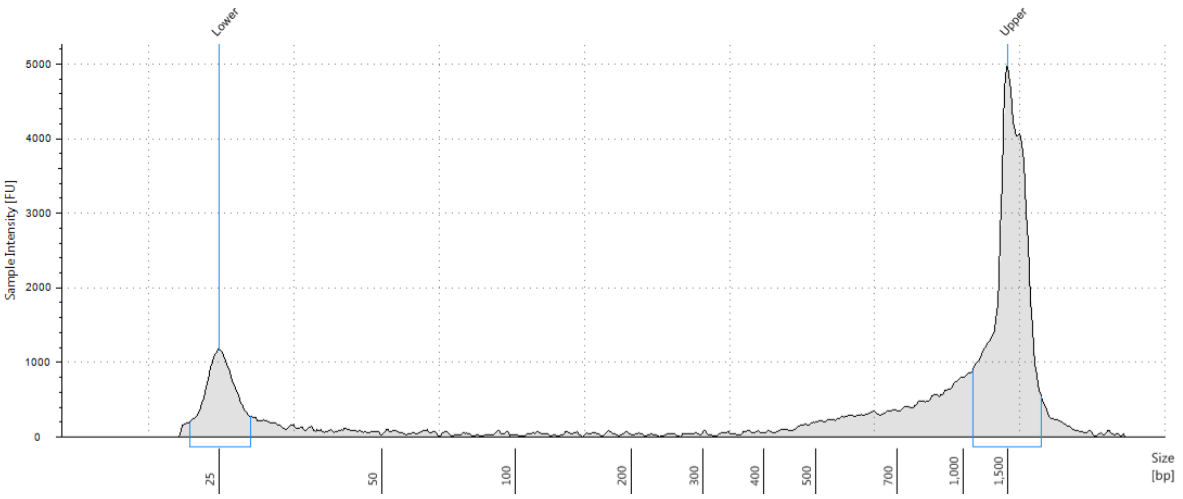

(L) Embryo ID 41HF

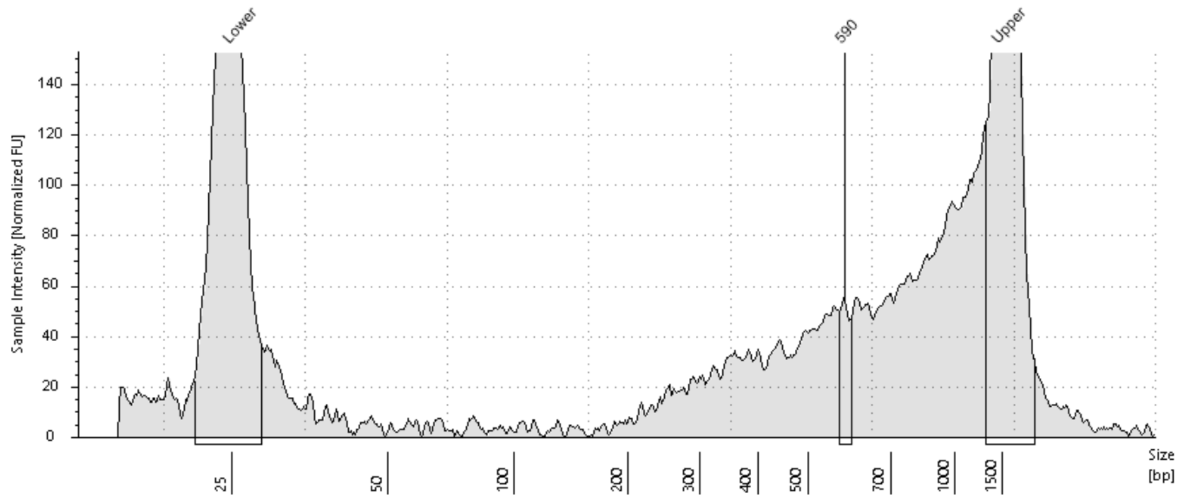

(M) Embryo ID 42HF

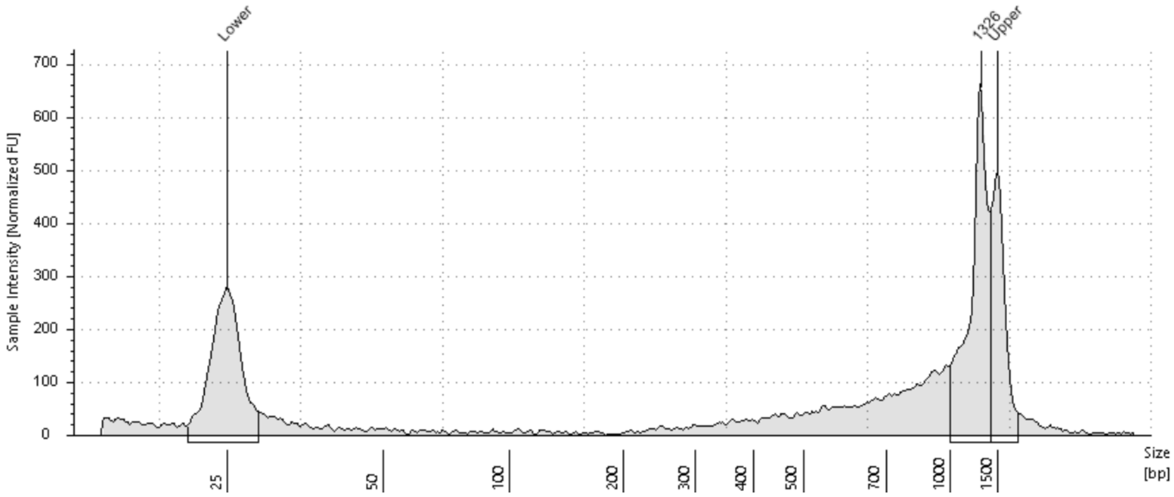

(N) Embryo ID 13LF

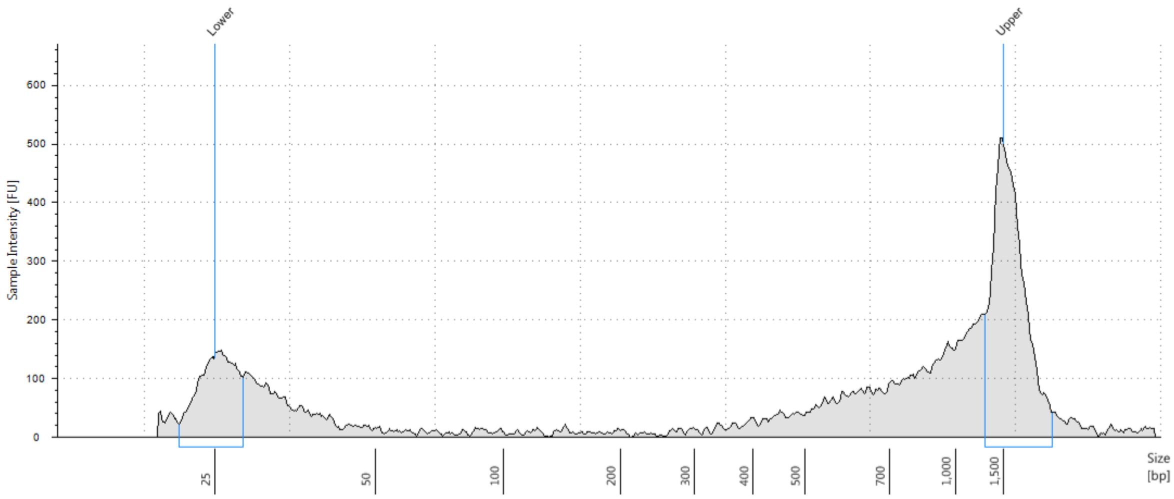

(O) Embryo ID 14LF

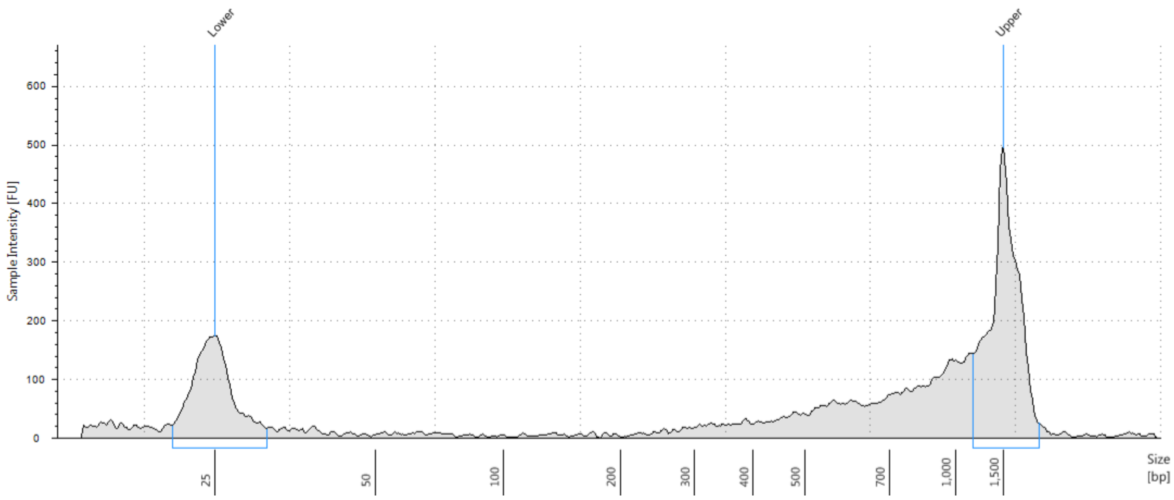

(P) Embryo ID 48LF*

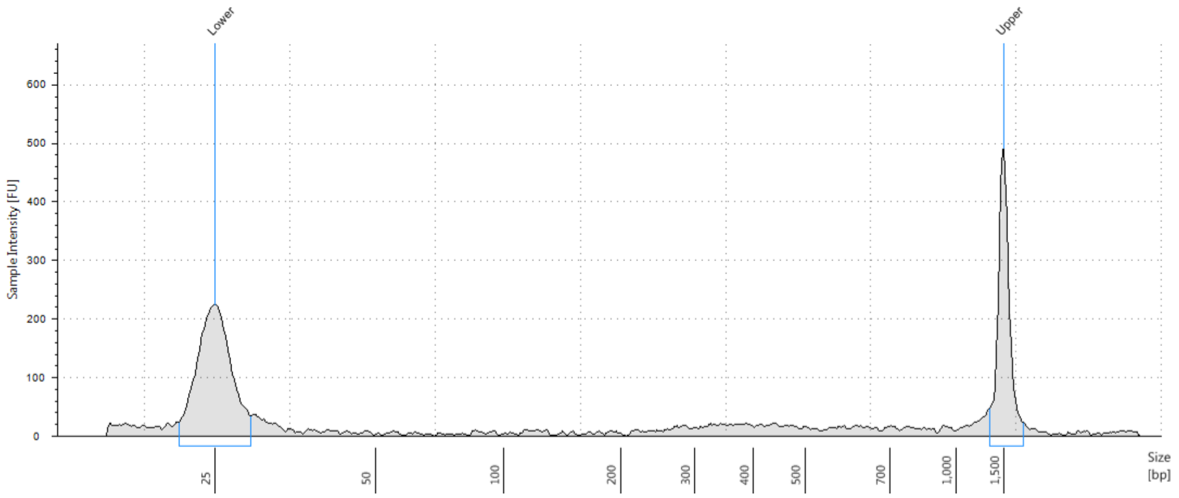

(Q) Embryo ID 49LF

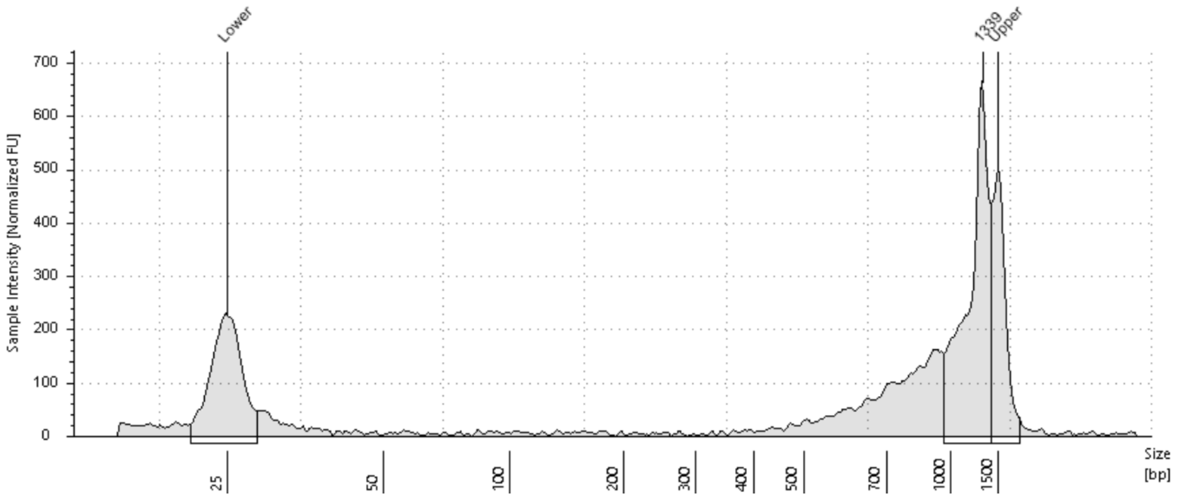

(R) Embryo ID 50LF

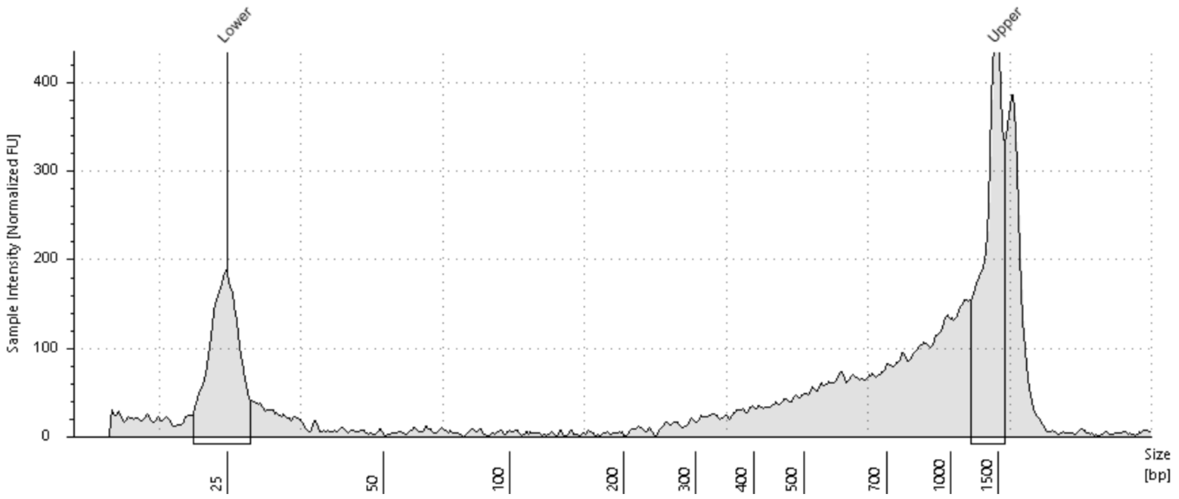

(S) Embryo ID 51LF

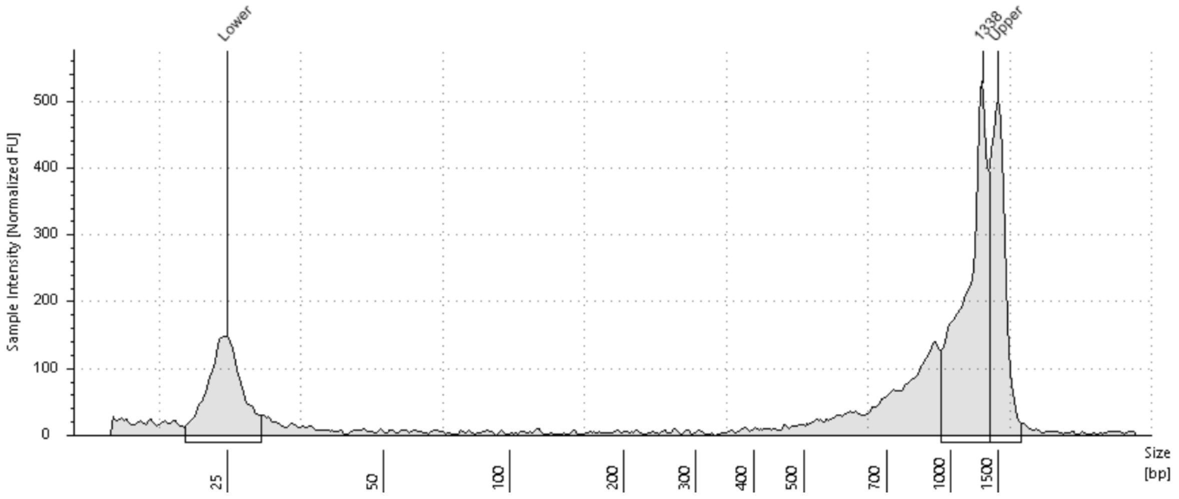

(T) Embryo ID 52LF

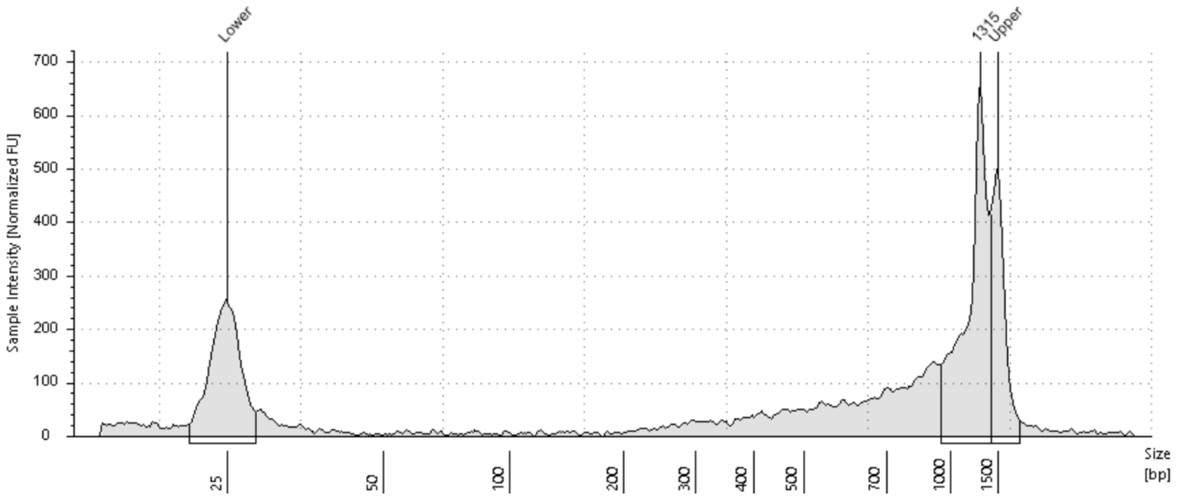

(U) Embryo ID 53LF

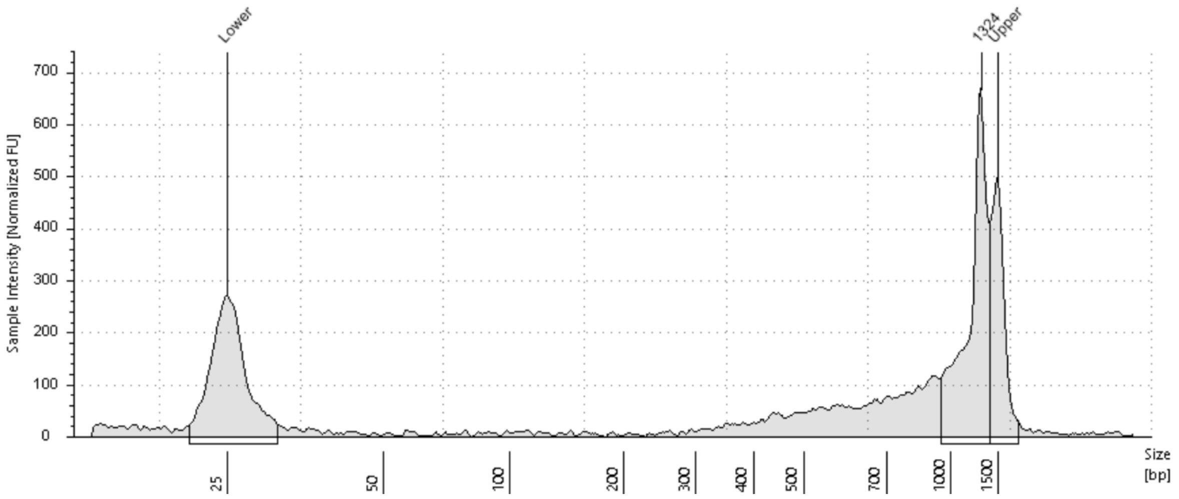

(V) Embryo ID 57LF

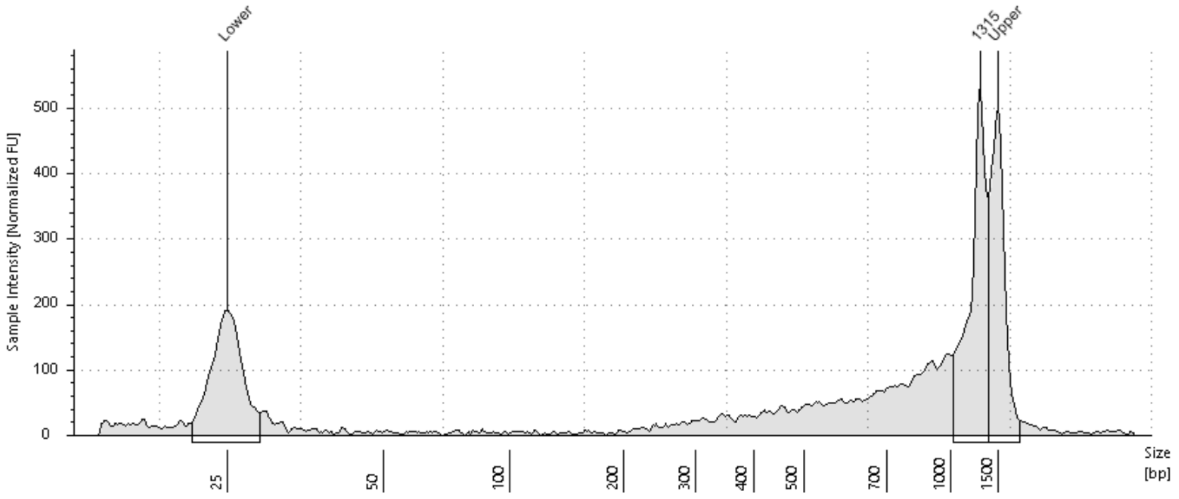

(W) Embryo ID 63LF

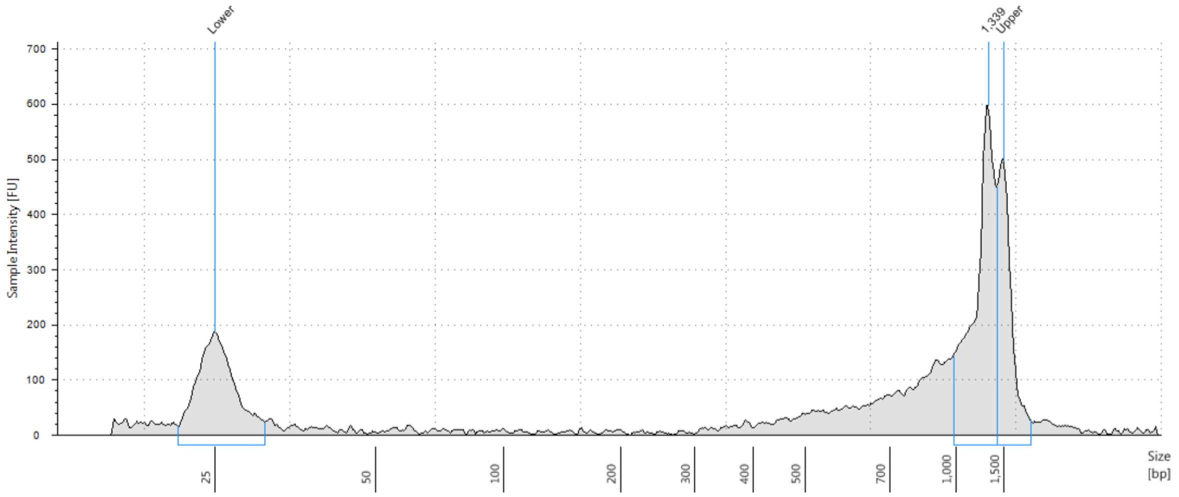

(X) Embryo ID 66LF*

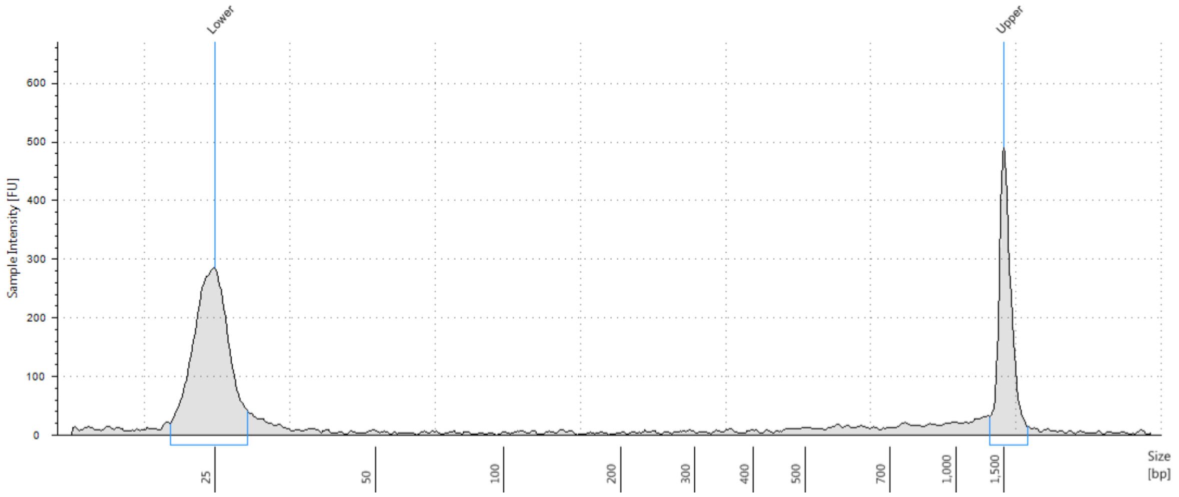

Supplement: Supplementary file 3 [file DataSheet1.PDF]
